# Identifying high-impact variants and genes in exomes of Ashkenazi Jewish inflammatory bowel disease patients

**DOI:** 10.1038/s41467-023-37849-3

**Published:** 2023-04-20

**Authors:** Yiming Wu, Kyle Gettler, Meltem Ece Kars, Mamta Giri, Dalin Li, Cigdem Sevim Bayrak, Peng Zhang, Aayushee Jain, Patrick Maffucci, Ksenija Sabic, Tielman Van Vleck, Girish Nadkarni, Lee A. Denson, Harry Ostrer, Adam P. Levine, Elena R. Schiff, Anthony W. Segal, Subra Kugathasan, Peter D. Stenson, David N. Cooper, L. Philip Schumm, Scott Snapper, Mark J. Daly, Talin Haritunians, Richard H. Duerr, Mark S. Silverberg, John D. Rioux, Steven R. Brant, Dermot P. B. McGovern, Judy H. Cho, Yuval Itan

**Affiliations:** 1 The Charles Bronfman Institute for Personalized Medicine, Icahn School of Medicine at Mount Sinai, New York, NY USA; 2 Department of Genetics, Yale University, New Haven, CT USA; 3 Department of Genetics and Genomic Sciences, Icahn School of Medicine at Mount Sinai, New York, NY USA; 4 Translational Genomics Unit, F. Widjaja Foundation Inflammatory Bowel and Immunobiology Research Institute, Cedars-Sinai Medical Center, Los Angeles, CA USA; 5 St. Giles Laboratory of Human Genetics of Infectious Diseases, The Rockefeller University, New York, NY USA; 6 Department of Neurology, Massachusetts General Hospital, Boston, MA USA; 7 Immunology Institute, Graduate School, Icahn School of Medicine at Mount Sinai, New York, NY USA; 8 St. Giles Laboratory of Human Genetics of Infectious Diseases, Rockefeller Branch, The Rockefeller University, New York, NY USA; 9 Department of Pediatrics, Cincinnati Children’s Hospital Medical Center, Cincinnati, OH USA; 10 Department of Pediatrics, University of Cincinnati College of Medicine, Cincinnati, OH USA; 11 Department of Pathology, Albert Einstein College of Medicine, New York, NY USA; 12 Division of Medicine, University College London (UCL), London, UK; 13 Research Department of Pathology, University College London (UCL), London, UK; 14 Moorfields Eye Hospital NHS Foundation Trust, London, UK; 15 Department of Pediatrics, Emory University, Atlanta, GA USA; 16 Institute of Medical Genetics, Cardiff University, Cardiff, UK; 17 Department of Public Health Sciences, University of Chicago, Chicago, IL USA; 18 Division of Gastroenterology, Hepatology and Nutrition, Oncology Boston Children’s Hospital, Boston, MA USA; 19 Institute for Molecular Medicine Finland (FIMM), University of Helsinki, Helsinki, Finland; 20 Medical and Population Genetics, Broad Institute, Cambridge, MA USA; 21 Analytical and Translational Genetics Unit, Massachusetts General Hospital, Boston, MA USA; 22 Division of Gastroenterology, Hepatology and Nutrition, Department of Medicine, University of Pittsburgh School of Medicine, Pittsburgh, PA USA; 23 Department of Human Genetics, University of Pittsburgh Graduate School of Public Health, Pittsburgh, PA USA; 24 Inflammatory Bowel Disease Centre, Mount Sinai Hospital, Toronto, Ontario Canada; 25 Research Center, Montreal Heart Institute, Montréal, Québec Canada; 26 Department of Medicine, Université de Montréal, Montréal, Québec Canada; 27 Division of Gastroenterology, Department of Medicine, Rutgers Robert Wood Johnson Medical School, New Brunswick, NJ USA; 28 Department of Genetics and the Human Genetics Institute of New Jersey, Rutgers University, Piscataway, NJ USA; 29 Meyerhoff Inflammatory Bowel Disease Center, Department of Medicine, Johns Hopkins University School of Medicine, Baltimore, MD USA; 30 Department of Medicine, Icahn School of Medicine at Mount Sinai, New York, NY USA

**Keywords:** Inflammatory bowel disease, Disease genetics, Medical genomics, Genomic analysis, Genome informatics

## Abstract

Inflammatory bowel disease (IBD) is a group of chronic digestive tract inflammatory conditions whose genetic etiology is still poorly understood. The incidence of IBD is particularly high among Ashkenazi Jews. Here, we identify 8 novel and plausible IBD-causing genes from the exomes of 4453 genetically identified Ashkenazi Jewish IBD cases (1734) and controls (2719). Various biological pathway analyses are performed, along with bulk and single-cell RNA sequencing, to demonstrate the likely physiological relatedness of the novel genes to IBD. Importantly, we demonstrate that the rare and high impact genetic architecture of Ashkenazi Jewish adult IBD displays significant overlap with very early onset-IBD genetics. Moreover, by performing biobank phenome-wide analyses, we find that IBD genes have pleiotropic effects that involve other immune responses. Finally, we show that polygenic risk score analyses based on genome-wide high impact variants have high power to predict IBD susceptibility.

## Abstract (web summary)

Inflammatory bowel disease (IBD) is highly prevalent among the Ashkenazi Jewish population. Here, the authors identify novel IBD-associated variants and genes, validated by transcriptomic and phenome-wide associations.

## Introduction

Inflammatory bowel disease (IBD) is a group of chronic diseases where sections of the gastrointestinal tract become inflamed due to an aberrant immune response to intestinal bacteria and microbiota in genetically susceptible individuals. The bulk of IBD cases comprise Crohn’s disease (CD) and ulcerative colitis (UC). Genome-wide association studies (GWAS) have identified more than 200 IBD risk loci to date, mostly in Europeans^1–4^. The Ashkenazi Jewish (AJ) population has a high IBD susceptibility, with a 2- to 4-fold increased risk of developing IBD due to an AJ founder effect and long-term genetic isolation^5–7^. A recent study indicated that 34% of rare protein-coding variants present in the AJ population are significantly enriched by comparison with other reference populations^8^. Therefore, rare and high-impact genetic variants in AJ may address and complement the missing heritability in current IBD GWAS studies of common genetic variants^9^.

In this study, we genetically identified 4453 QC-passed AJs of 1734 cases and 2719 controls from whole exome sequencing (WES) data of the NIDDK IBD Genetics Consortium (IBDGC). We employed several cutting-edge approaches to select highly plausible rare variants predicted to have high phenotypic impact, and then performed a SNP-set Kernel Association Test (SKAT) on gene-level aggregations of these variants. In addition, we performed meta- and pathway enrichment analyses to identify novel plausible IBD-causing candidate genes whose biological plausibility was further assessed by bulk RNA sequencing (RNA-seq) and single-cell RNA sequencing (scRNA-seq) analyses. Additionally, we performed gene-level phenome-wide association study (PheWAS) analyses to explore shared risk genes associated with other diseases in Mount Sinai Hospital’s BioMe BioBank. Finally, we tested the polygenic risk score (PRS) classification performance of predicted high-impact variants in IBD cases and unaffected controls by using machine learning and a deep learning classifier.

## Results

**Fig. 1:**
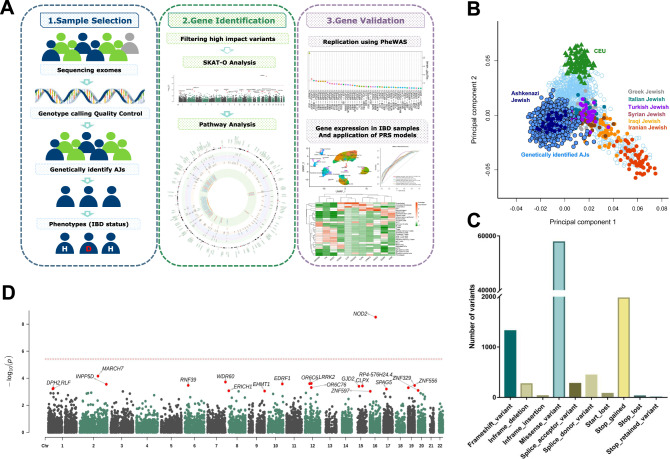
Ashkenazi Jewish population-specific case-control study using high-impact variants revealed IBD-associated genes. **A** Flowchart of present work. Firstly, AJ samples from all WES participants that passed quality control were genetically identified. Then, high impact rare variants from exomes were filtered using cutting-edge mutation filtering approaches; high impact rare variants were aggregated into gene sets to perform SKAT-O gene burden analyses on IBD cases and controls of AJs. IBD associations were validated and prioritized at the pathway level, gene level, and variant level using multiple methods. Next, we replicated the top candidate genes in an independent cohort and identified their relatedness to diseases other than IBD using gene-level PheWAS. Then, gene expression was tested in both bulk RNA-seq and single-cell RNA-seq. Lastly, we built polygenic risk score models to predict IBD patients using high-impact variants. **B** We filtered a set of LD-pruned independent sites to perform a fastSTRUCTURE admixture analysis by comparing individual samples with 36 known AJ reference samples. The lowest AJ fraction (0.645) in the AJ reference panel was used as the threshold, above which a WES sample was deemed to be genetically AJ and retained for further analyses. Genetically identified Ashkenazi Jewish samples are displayed on a PCA plot compared to the Jewish and European reference panels. The genetically identified AJs constitute an independent cluster, which overlapped with the AJ reference panel but was distinct from the European cluster. **C** Distributions of filtered high impact rare variants by molecular function. **D** SKAT-O analysis on 1734 AJ IBD cases and 2719 AJ controls. The red dashed line indicates the Bonferroni-adjusted *P* values of genome-wide significance. All dots represent negative log unadjusted *P* values.

**Fig. 2:**
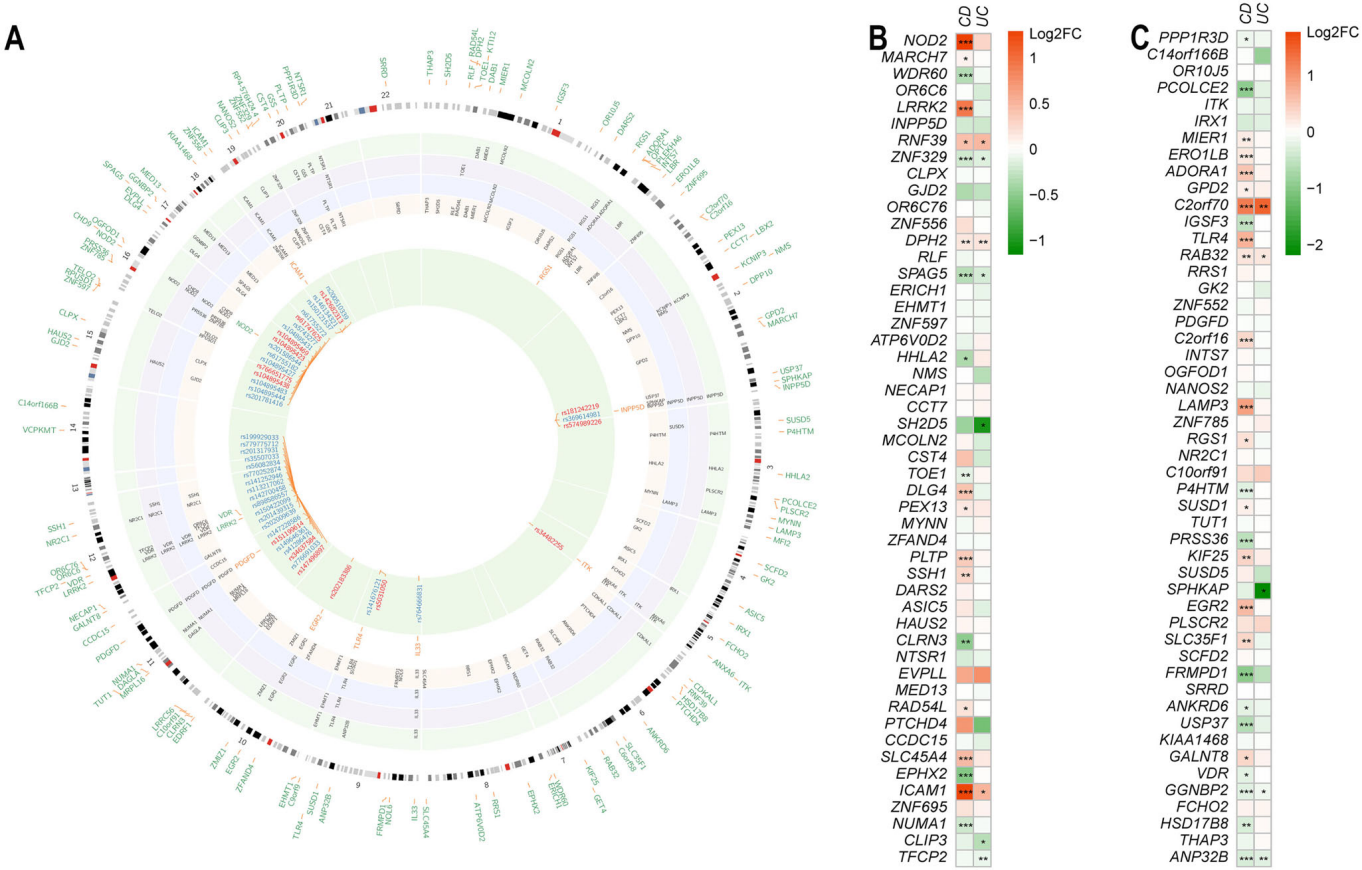
Pathway approaches prioritized IBD-associated genes derived from case-control studies, and differential expression of IBD-associated genes in CD, UC vs. controls. **A** A Circos plot summarizes the process of identifying IBD-associated genes and variants. The outer layer includes all 127 SKAT-O-derived IBD candidate genes with *P* < 0.01. The intermediate four layers represent the top genes identified by four different pathway enrichment and biological relatedness analyses (green: ToppGene; purple: Human Gene Connectome; blue: GIANT; yellow: Ingenuity Pathway Analysis). The 11 genes listed between the intermediate layers and the inner green layer are significant gene loci identified in common by all four pathway approaches (the three known IBD genes are in green, the 8 novel genes in orange). The inner layer displays all 46 high-impact rare variants in the 11 orange genes. Of the 46 variants, 14 variants (highlighted in red inside the inner green layer) are associated with IBD (*P* < 0.05). We also prioritized the candidate IBD genes resulting from the SKATO test by combining all pathway and functional module analysis results (“Methods”, Supplementary Data 6 and Supplementary Fig. 7). *EGR2*, *ICAM1*, *IL33*, *INPP5D*, *ITK*, *LRRK2*, *NOD2*, *TLR4, VDR* are more significant as they remained among the top 10% IBD-associated genes for both the SKATO test and biological function prioritization. **B** Log-fold changes of the top 50 genes derived from IBD SKAT-O analysis in CD, UC *vs*. controls bulk RNA-seq analyses (*P < 0.05; **P < 0.01; ***P < 0.001, same levels for **C**). **C** Log-fold changes of the top 51–100 genes derived from IBD SKAT-O analysis in CD, UC *vs*. controls bulk RNA-seq analyses. All statistical tests are two-sided. Exact *P* values are provided in Supplementary Data 12.

To generate a homogeneous and genetically matched dataset of cases and controls, we first genetically identified 4453 samples of AJ ancestry across 9076 QC-passed IBDGC WES samples, comprising 1734 IBD AJ cases (1138 CD, 458 UC, and 138 IBD) and 2719 AJ controls, the largest genetically identified AJ WES cohort to date. We then performed a SNP-set Kernel Association Test (SKAT)^10^ on gene-level aggregations of filtered high-impact variants, obtained by integrating effective complementary variant- and gene-level approaches to select highly credible deleterious variants (Fig. 1a, b, Supplementary Fig. 1 and Fig. 2). We performed an AJ IBD case-control optimized SKAT (SKAT-O) analysis on 13,628 genes harboring 63,864 high impact rare variants (Fig. 1c), and then performed SKAT-O analyses of IBD, CD and UC cases versus unaffected controls. We identified one gene, the well-characterized CD gene *NOD2*, that displayed genome-wide significance (Bonferroni-corrected *P* = 3.76 × 10^−6^ (= 0.05/13,268), Fig. 1d, Supplementary Figs. 3, 4 and Supplementary Data 1–3). To examine the contribution of variants within the significant genes, we performed a logistic regression association analysis on all high-impact variants comparing AJ IBD cases to AJ unaffected controls (Supplementary Data 4, Supplementary Fig. 5) for variants with *P* < 0.05 and their host genes.

**Fig. 3:**
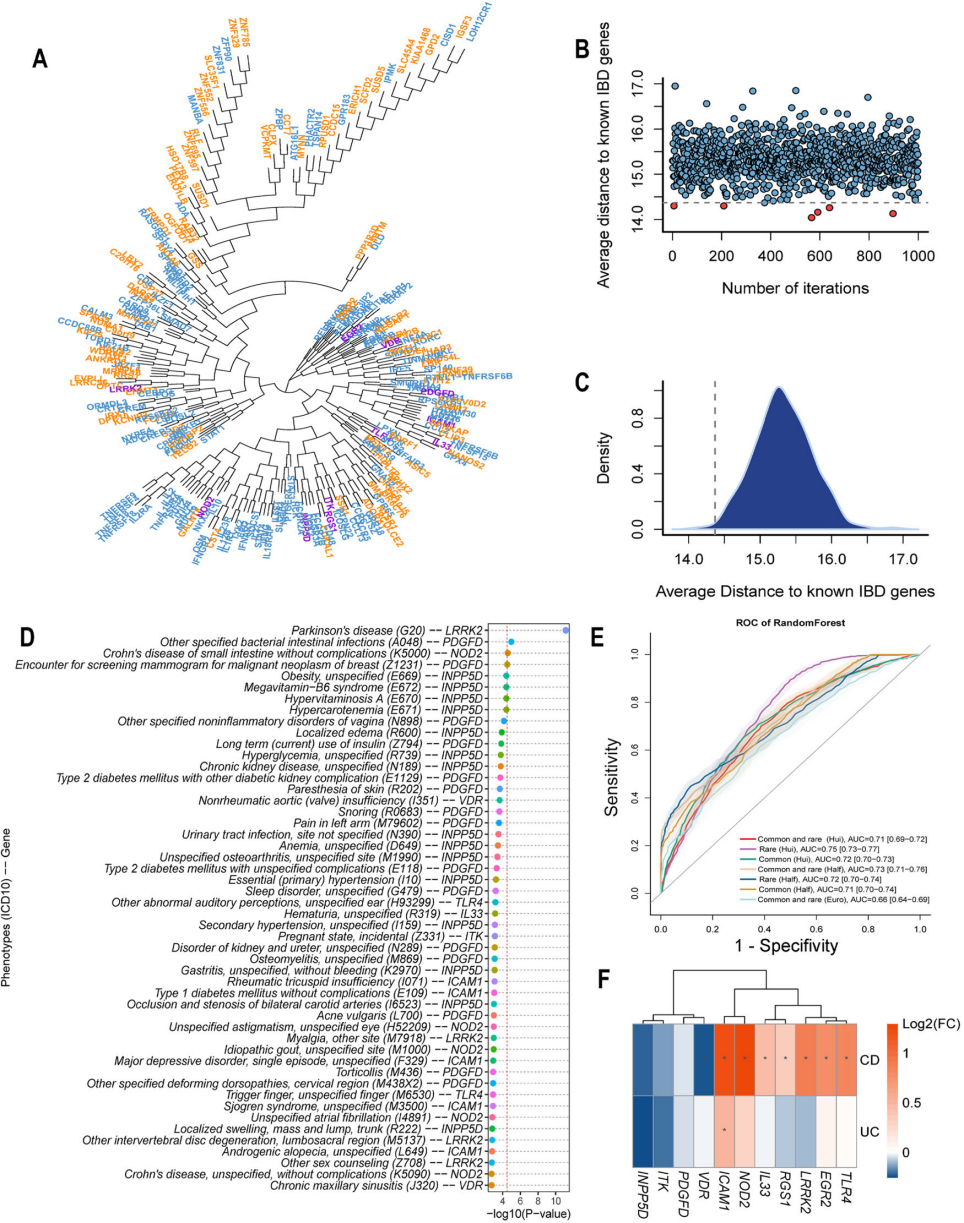
IBD candidate genes are biologically proximate to IBD known genes. Gene level PheWAS replicated IBD-associated genes in an independent cohort and demonstrated that IBD genes are also related to other immune responses. **A** Clustering IBD candidate genes (blue) and IBD known genes (orange) according to biological relatedness by Functional Genomic Alignment function using the HGC. The 11 pathway identified genes (Fig. 2A) are indicated in violet. The candidate genes did not form distinct clusters; rather, they were mixed with IBD known genes. **B** A dot plot representing the average distance from a randomly selected gene set (127 genes) to known IBD genes. The gray dashed line represents the cutoff in terms of the average biological distance between IBD-associated genes and IBD known genes. With genes randomly resampled 1000 times, 6 random gene sets have lower average distances; hence, the empirical *P* value of our candidate genes being empirically associated with IBD as a group is 0.006. *P* value is unadjusted and the statistical test is two-sided. **C** A density plot for all average distances in a resampling test; the percentiles at 2.5% and 97.5% are 14.27 and 14.48, respectively. The vertical dashed line denotes the cutoff in plot (**B**). **D** Gene level PheWAS analysis on the 11 candidate genes using 40 K whole exome sequencing samples from the Mount Sinai BioMe Biobank. The top 50 associations are displayed, the red dashed line denotes two-sided unadjusted *P* = 0.01 in the gene level PheWAS. **E** Comparisons of prediction results on IBD individuals using polygenic risk scores derived from different variant sets. Receiver Operating Characteristic (ROC) curves of PRS derived from different variant sets (solid lines), and the 95%CI (bands). Names in brackets indicating GWAS summary statistics used: Hui, AJ IBD GWAS from Hui’s study^38^; Half, AJ IBD GWAS using half of our IBDGC samples; Euro, European IBD GWAS from Liu’s study^2^. Values indicate the estimated AUCs and their 95% confidence intervals (in square brackets). **F** RNA-seq log2 fold change of the 11 IBD-associated genes identified by all four pathway analyses in CD, UC, and IBD versus controls, respectively (*P < 0.05). *P* values are unadjusted and statistical tests are two-sided. Exact *P* values are provided in Supplementary Data 12.

Since biologically-relevant genes might not display genome-wide significance at the gene level due to genetic heterogeneity, we additionally applied pathway enrichment and biological relatedness approaches to identify biologically plausible IBD-associated genes that we obtained from the SKAT-O results. The significance cut-off in the AJ IBD SKAT-O test was relaxed to *P* < 0.01 in order to capture other possible IBD-associated candidates (127 genes, of which 3 are already known IBD-associated genes: *LRRK*2, *NOD2*, and *VDR*)^4^, from which a subset of genes was prioritized by 4 complementary pathway enrichment and biological relatedness methods: Ingenuity Pathway Analysis (IPA), ToppGene, Genome-Scale Integrated Analysis of Networks in Tissues (GIANT), and Human Gene Connectome (HGC) (Fig. 2a, “Methods”). To estimate the functional relevance of the candidate genes to IBD, we calculated their average HGC biological proximity to 157 known IBD-causing genes (Supplementary Data 5) and compared it to equivalent sets of randomly sampled genes in 10,000 resampling iterations, obtaining *P* = 0.0072 (Fig. 3b, c and Supplementary Fig. 6), thereby demonstrating a significant functional association of the SKAT-O list of candidate genes to IBD. In addition, we applied functional genomic alignment (FGA)^11^ to cluster all candidate genes with known IBD genes by their biological distance (Fig. 3a). The candidate genes were evenly intermixed with the known IBD genes. These results indicate that the candidate IBD genes are indeed likely to be associated with the IBD phenotype (Supplementary Data 6, 7).

We identified a final list of 11 genes (*EGR2, ICAM1, IL33, INPP5D, ITK, LRRK2*, *NOD2*, *PDGFD, RGS1, TLR4*, and *VDR*) that occurred within the top-ranking results across all 4 pathway enrichment and biological relatedness approaches (IPA, ToppGene, GIANT and HGC). All of these genes have been reported as having pathogenic mutations in non-IBD diseases (*ICAM1*, *ITK*, *LRRK2, NOD2*, and *INPP5D* in primary immunodeficiency; *EGR2* in systemic lupus erythematosus; *PDGFD, IL33,* and *RGS1* in autism spectrum disorder; *TLR4* in type 2 diabetes, gastritis and susceptibility to infectious diseases; *VDR* in Vitamin D-resistant rickets) in the Human Gene Mutation Database (HGMD) Professional version^12^. In summary, three genes, *NOD2, LRRK2*, and *VDR* are known IBD genes (Supplementary Data 7), whereas the other 8 are novel, and not yet formally implicated in IBD (Fig. 2, Supplementary Results).

We then investigated the likely physiological relatedness of the 8 novel prioritized genes to IBD. Variants in *ICAM1* and *INPP5D* are reported to be associated with primary immunodeficiencies in HGMD. *ICAM1* is involved in mediating adhesive interaction between lymphocytes and endothelial cells, and has been recognized as a potential therapeutic target in IBD^13,14^. Since *ICAM1* is located within 100 kb of *TYK2* (a gene known to be associated with IBD pathogenesis^15^, we sought to determine whether the *ICAM1* lead variant (rs142682313, OR = 0.4, *P* = 7.16 × 10^−4^) was conditionally independent of IBD-associated sites in *TYK2*. To this end, we performed Genome-wide Complex Trait joint and conditional analyses (GCTA-COJO)^16^ with the *ICAM1* lead SNP and three IBD-associated sites in *TYK2*, both of which suggested that the *ICAM1* IBD variants act independently of the *TYK2* variants. One of the *TYK2* IBD variants, rs12720356, remained as an IBD-associated variant in the AJ cohort based on joint association analysis (Supplementary Data 8 and 9). *INPP5D* encodes SHIP1 protein, whose expression level is significantly associated with IBD^17,18^. *INPP5D* resides in close proximity on chromosome 2q37.1 to another IBD gene, *ATG16L1*^19^. We therefore performed linkage disequilibrium (LD) analysis on the most significant variant in *INPP5D*, rs574989226, and demonstrated that there were no strong LD pairs identified between *ATG16L1* and *INPP5D* (Supplementary Data 10). Additionally, we performed a conditional analysis on the well-described IBD variant rs2241880^20^ in *ATG16L1* to check the independence of rs574989226. The significance of rs574989226 only slightly changed after the GCTA-COJO conditional test using our AJ cohort (conditioned, *P*_con_ = 1.29 × 10^−2^; unconditioned, *P*_uncon_ = 1.04 × 10^−2^ from GCTA-COJO), which indicates that rs574989226 is independent from rs2241880.

Variants in *EGR2* are associated with the autoimmune diseases, systemic lupus erythematosus, and celiac disease. As a member of a zinc finger transcription factor family, *EGR2* is known to display suppressive activity with regard to CD4^+^ T cells, and control the production of inhibitory cytokines such as IL-10 and TGF-β1^21^. A previous study also revealed that the expression of *EGR2* is upregulated in inflamed colonic biopsies when compared to healthy colon^22^, suggesting that *EGR2* is likely to be an IBD-associated gene. *IL33* has long been considered to play an important role in intestinal immunity. *IL33* and its membrane receptor *ST2* act as critical regulators of inflammation^23,24^. *TLR4* plays a key role as the hub of the immune response to microbes in the gut in IBD pathogenesis^25^. *PDGFD*, a differentially expressed gene in crypt-associated fibroblasts, has been reported to be significantly downregulated in the colonic mucosa of Crohn’s disease patients^26^. Moreover, single-cell analyses of Crohn’s disease tissues revealed that γδ T cells selectively expressed *PDGFD*^27^, indicating that *PDGFD* might play a role in IBD. Although there are no records of IBD for *RGS1* in HGMD, *RGS1* is a member of the regulators of G-protein signaling (RGS) family, which is considered to be a promising target for the treatment of gastrointestinal inflammation^28^. Gibbons et al. have shown that *RGS1* expression is significantly higher in human gut T cells compared to T cells derived from peripheral blood and this difference can further increase with intestinal inflammation. More specifically, *RGS1* mRNA is significantly elevated in T cells obtained from intestinal samples of CD and UC patients when compared with healthy controls. They have also demonstrated that RGS1 is a dominant regulator of T cell trafficking in the gut, and therefore it could be involved in the pathology of IBD^29^.

IL-2-inducible tyrosine kinase (*ITK*) is primarily expressed in T cells, and is essential for proximal T cell receptor (TCR) signaling. Studies have shown that *ITK* is involved in the pathogenesis of autoimmune diseases, including rheumatoid arthritis, systemic lupus erythematosus, multiple sclerosis, and IBD^30^. *ITK* harbors a variant (rs753847568, p.Val264Ile) associated with very early onset inflammatory bowel disease (VEO-IBD) according to the HGMD^31^. Since five of the identified IBD-associated genes have been implicated in primary immunodeficiency, which is closely linked with VEO-IBD, we used HGC to check the biological association of candidate genes with the list of known VEO-IBD-causing genes^32^. The analysis of known VEO-IBD-causing genes versus random gene sets yielded a *P* = 0.023 in 10,000 resampling iterations. Interestingly, these analyses indicated that the genetic basis of AJ IBD resembles that of IBD in young children under the model built using rare high-impact mutations. Taken together, these findings demonstrated the strength of population-specific analyses in AJ. Therefore, all 8 novel genes described in this study are likely to have functional relevance to IBD.

Investigating the two well-known IBD genes prioritized in our analyses, *NOD2* had higher significance in the CD-specific SKAT-O analysis (*P* = 9.51 × 10^−14^_,_ Supplementary Fig. 3 and Supplementary Data 2) but was insignificant in the UC-specific analysis (*P* = 0.85) (Supplementary Fig. 4 and Supplementary Data 3) as expected, since *NOD2*^33^ is not known to cause UC. The significance of *LRRK2* demonstrated the same trend: *LRRK2* was more significant in the CD-specific test (*P* = 9.68 × 10^−4^) and the IBD-specific test (*P* = 2.53 × 10^−4^), but showed no significance in the UC-specific test (*P* = 0.07). However, *LRRK2* showed lower significance compared to *NOD2*, due to the number of nominal significant *LRRK2* variants that were used in the SKAT-O test (*LRRK2* has only one significant site among 14 high-impact variants, whereas *NOD2* has five significant sites among 14 high impact variants, see Supplementary Data 11). The *NOD2* rs104895438 and *LRRK2* rs34637584 variants have been shown to be enriched in the AJ population and their independence has been confirmed by a previous study^8^ via conditional analyses, whereas the other variants have not previously been implicated in IBD.

These 11 plausible IBD candidate genes harbor a total of 46 high-impact variants (Supplementary Data 11). To test the burden of the significant SNPs (*P* < 0.05) located within the IBD candidate genes, we aggregated all significant SNPs from each IBD candidate gene into a single SNP set; the mutation carrier frequency in cases was 15.74% compared to 9.26% in controls, with an odds ratio (OR) of 1.83 (*P* = 8.78 × 10^−11^ by chi-squared test) despite two protective sites that are included in the analyses.

**Fig. 4:**
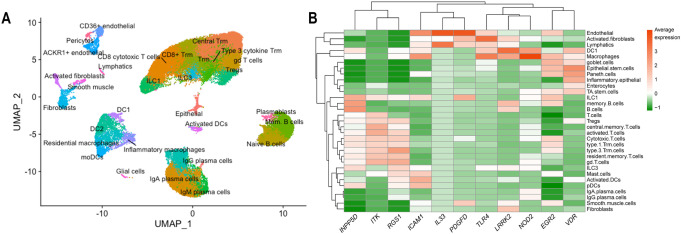
Single-cell RNA-seq analyses indicate that the nine IBD genes identified in this study are expressed in ileum from Crohn’s disease cases. **A** UMAP plot of clustered scRNA-seq cells from ileal CD inflamed and uninflamed samples which can be annotated broadly into 31 cell types. **B** scRNA-seq average expression of the 11 genes identified from pathway analyses across the 31 cell types. For each gene, the non-zero expressed cells were utilized to calculate the average expression in each cell type. The average expression levels in all cell types were scaled for comparison.

We then performed bulk RNA-seq analyses in IBD (CD and UC) patients *vs*. unaffected controls^34^, and found that 7 (*ICAM1*, *NOD2*, *IL33*, *RGS1*, *LRRK2*, *EGR2*, and *TLR4*) of the 11 candidate genes are significantly over- or under-expressed in either CD or UC (“Methods”, Figs. 2b, c and Fig. 3f, Supplementary Data 12). With the exceptions of *INPP5D*, *ITK, VDR*, and *PDGFD*, which have lower expression levels in both CD and UC cases, all genes exhibiting increased expression displayed higher expression levels in CD or UC. We performed pathway analysis for the 127 significant genes (*P* < 0.01) identified by SKAT-O and weighing the significant genes based on their log fold change and *p* values obtained from bulk RNA-seq analyses. Among the results of related ‘Disease and Disorder’ analysis by IPA, the ‘Cancer’, ‘Organismal Injury and Abnormalities’, and ‘Gastrointestinal Disease’ were the top 3 mostly related disorders. Additionally, we tested gene expression data from scRNA-seq analyses of CD samples^35^, where the clustering of 70,226 cells from 11 paired samples (inflamed and uninflamed biopsies obtained from surgically resected ileal tissues) resulted in 36 clusters that could be annotated broadly into 31 cell types based on the expression of specific cell type markers (Fig. 4a). We examined the expression of the 11 IBD candidate genes in the 31 different cell types in the ileum. Generally, each of the 11 genes displayed over-expression in at least one cell type. *PDGFD* and *IL33* were mostly expressed in endothelial cells, whereas the other 9 genes showed expression in different immune system cells (Fig. 4b and Supplementary Data 12). In total, the upregulated genes (*TLR4*, *EGR2*, *LRRK2*, *NOD2*, and *ICAM1*) identified from bulk-RNA-seq also displayed over-expression in macrophages and dendritic cells (DC1) (Supplementary Fig. 8), suggesting that these genes are likely to be involved in immune activation and response.

We also tested IBD associations by performing a meta-analysis on two IBDGC datasets. Single variant associations were analyzed by raremetalworker, which provided summary statistics for the gene-level meta-analysis in RAREMETAL (“Methods”, Supplementary Data 13 and 14). Of the 13,289 genes that were investigated, only two genes, *ZSCAN5B* and *NOD2*, passed the Bonferroni-corrected threshold of *P* < 3.76 × 10^−6^ in IBD case-control meta-analysis (Supplementary Data 15). However, the function of *ZSCAN5B* in IBD has yet to be investigated in future studies. Following *NOD2*, although not passing Bonferroni-adjusted significance, *BIN3* and *DAGLA* have relatively strong association with IBD. *BIN3* is a tumor suppressor gene which, interestingly, has been found to be upregulated in the healed mucosa of UC patients when compared with non-healed inflamed mucosa^36^. *DAGLA* was suggested as a potential druggable target based on its increased expression in ulcerative pancolitis compared to healthy human colonic tissue^37^. *ICAM1*, *LRRK2, NOD2*, *PDGF*, and *RGS1* were again identified as IBD-associated genes at the same significance level (*P* < 0.01) in the meta-analysis.

To investigate whether the 11 candidate genes can be replicated in an independent cohort and to explore their involvement in other traits, we conducted gene-level phenome-wide association studies (PheWAS) utilizing genotype and phenotype data compiled in Mount Sinai’s EMR-linked BioMe BioBank. As a result, the IBD association of *NOD2* and *ICAM1* was replicated in gene-level PheWAS. Moreover, these genes likely have pleiotropic effects across other phenotypes (Supplementary Results, Methods, Fig. 3d and Supplementary Data 16). Lastly, we evaluated the performance of rare and high-impact variants in identifying individuals at risk for IBD using PRS with a Random Forest machine learning classification algorithm. We first used LD-pred to calculate the polygenic risk score for each individual, then employed risk scores as features to predict the IBD status of individuals with a Random Forest machine learning algorithm. We compared models based on the risk score results from six combinations of two GWAS summary statistics (a previous GWAS on AJ IBD samples from Hui et al.^38^; the other GWAS on half of our AJ IBD samples) and three sets of variants (high impact rare sites, common variants and both sets combined). As shown in Fig. 3e, the combinations of GWAS summary statistics and SNP sets displayed comparable predictive power, which result in areas under the curve (AUC) ranging from 0.71 to 0.75, whilst high impact rare variants displayed slightly better predictive power compared to common variants under both GWAS statistic sets. The AUC of rare variants were 0.75 and 0.72 for Hui et al. and our GWAS statistics, respectively, whereas the AUC of common variants were 0.72 and 0.71, respectively. The integrated discrimination improvement (IDI) calculated by PredictABEL^39^ was used to evaluate risk predictions from the model with common variants and the model with both common and rare variants. Using both common and rare variants in the model improved reclassification with an IDI of 1.00% for Hui’s GWAS summary statistic, but decreased the reclassification with an IDI of 2.69% for the GWAS summary statistics generated by half of our dataset, which indicate that the effects of rare variants may require accurate assessment in risk prediction. As an alternative PRS method for comparison, we built a deep learning model to predict high-risk individuals using the same high impact rare sites as the above model. We trained a 7-layer convolutional neural network (CNN) model, which yielded an AUC of 0.69 in 5-fold cross-validation. The power of the deep learning approach was likely restricted by the available sample size, and our sample size may be better suited for machine learning approaches such as Random Forest. The results indicate that high impact rare variants can provide predictive power that is equivalent to or better than common variants in identifying individuals at high risk for IBD, though common and rare variants may have different impacts on susceptibility to IBD. To further evaluate whether the population-specific PRS is more suitable for identifying risk of IBD among AJs, we adopted a non-AJ European-derived IBD GWAS statistics to generate PRS for the same half of our AJ samples. As a result, the AUC decreased from 0.73 to 0.66, indicating that the population-specific GWAS summary was more powerful for predicting the IBD risk.

In conclusion, we have conducted the first large-scale study of rare and high-impact genetic architecture in AJ IBD patients. The SKAT-O analyses yielded 127 significant IBD genes, that we further prioritized with pathway enrichment and biological relevance approaches, identifying 11 plausible IBD candidate genes, of which 8 are novel and two are well-established IBD genes. We further validated these candidate genes by RNA-seq and scRNA-seq analyses. Fourteen high-impact variants within these genes were identified as significant novel and plausible IBD-causing variants. We found that adult IBD under the rare and high impact genetic architecture displays similar genetic signals as VEO-IBD. PheWAS analyses on Mount Sinai Hospital’s BioMe BioBank samples revealed potential relatedness to IBD and other complex traits. Moreover, we employed high impact rare variant-derived PRS analyses to differentiate IBD cases from healthy controls, which displayed promising power to identify individuals at risk of IBD. These findings provide new insights into the etiology of rare and high-impact mutations underlying inflammatory bowel disease in the Ashkenazi Jewish population.

## Methods

### Sample collections

The NIDDK IBD Genetics Consortium (IBDGC) recruited samples through the following research centers: Cedars Sinai Medical Center, Icahn School of Medicine at Mount Sinai, Montréal-Boston Collaborative IBD Genetic Research Center, Johns Hopkins Genetic Research Center, University of Pittsburgh, University of Toronto and University College London, and a subset of Jewish controls from Cedars-Sinai Medical Center were obtained from The National Laboratory for the Genetics of Israeli Populations at Tel-Aviv University. Samples were collectively sequenced at the Broad Institute. We received a total of 9076 samples across two dataset releases (3822 and 5254, respectively), 54.3% of samples are male. Samples consisted of mixed populations, but the majority were broadly of European descent.

### Reads mapping and genotype calling

The raw sequence reads (Fastq files) were mapped to the reference genome by the Burrows-Wheeler Alignment (BWA) tool^40^. Mapped reads were passed to GATK to mark duplicated reads and were sorted in BAM format^41^. Next, local realignment was conducted around indels to clean up ‘SNP-like’ artifacts caused by mismatching bases introduced by alignments on the edge of indels. Lastly, the base quality score quality recalibration (BQSR) was performed to recalibrate inaccurate/biased quality estimates provided by the sequencing machine. The recalibrated bam files were passed to the variant discovery process to obtain highly credible variants stored in VCF files. During this procedure, any potential variants were called by HaplotypeCaller in GATK with GVCF model and gVCFs of single samples were merged into a single gVCF to perform GATK joint calling, which generates a raw VCF including all variants and indels. Finally, the variant quality score recalibration (VQSR) was applied to raw VCF to generate a new VCF containing high-quality variants calls. VQSR has two main steps; the first uses machine-learning to assign a well-calibrated probability to each variant in raw VCF. This score is then used as cutoff to extract high-quality variants. This process is run in two iterations with the SNP model and indel model, respectively. The final VCF is used for downstream analyses.

### Quality control

Several quality control processes were employed to ensure high-quality genotypes, and samples were used in the SKAT-O analysis. Specifically, at the variant level, variant sites were considered high-quality if they met the following criteria: (1) variants with a PASS filter status by Variant Quality Score Recalibration (VQSR). (2) variants with an average depth (DP) ≥10 and a genotype quality (GQ) ≥20 in all samples. (3) variants with an alternate allele that had a DP ≥10 and a GQ ≥20 in at least one individual. (4) variants with a ‘PASS’ value in the FILTER column of the gnomAD v2.1 VCF file. At the sample level, we further excluded any samples falling outside of 4 median absolute deviations (MAD) from the median for any of the given metrics^42^: (1) the ratio of the number of heterozygous genotypes to the number of homozygous alternate genotypes; (2) the transition/transversion ratio of the passing bi-allelic SNP calls made at dbSNP sites. (3) The insertion/deletion ratio of the indel calls made at dbSNP sites. This sample-level filtration step removed 101 outlier samples from the dataset. Samples were also excluded under the following criteria: greater than 3% missing genotypes; discordance between inferred gender based on genotype and self-reported gender; duplicated samples as identified by KING^43^; proportion of samples identical by descent >0.185. In addition, principal components were calculated (PLINK1.9^44^) and samples were removed if they were found to be statistically lower than the specific Ashkenazi Jewish proportion (details are described in ‘PCA and STRUCTURE analyses’ section). Variants were removed on the basis of the following criteria for the association tests: MAF > 1%, only rare variants were retained for high impact variants aggregating burden analysis; significant difference between missingness in cases compared with controls (*P* < 1.0 × 10^−5^); genotype rate <95% across samples, low average depth, extreme deviation from Hardy–Weinberg equilibrium (*P* < 1 × 10^−6^). Sample-level QC metrics including the percentage of aligned reads passing Illumina’s filter and the mean target coverage were obtained using Picard tools (https://broadinstitute.github.io/picard/), which revealed similar results for these measures in the two datasets (Supplementary Data 17). Finally, we checked the average depth of the samples for the overlapping variants in the two datasets to further evaluate if there was a remaining batch effect. There was no significant difference between the two datasets in terms of the average depth of samples (two-samples *t* test, *P* = 0.72). All quality control filtering was performed using PLINK1.9^44^ and R.

### RNA-seq data analyses

Full biopsies from the terminal ileum were collected from 302 newly diagnosed individuals under the age of 17 from the RISK cohort (GEO accession GSE57945)^45^. Samples were barcoded up to 12 per lane and sequenced using the Illumina HiSeq 2000. RNA-seq reads were mapped using TopHat2^46^ (to the human reference genome version 19). Approximately 20 million reads were successfully mapped for each individual. Following RNA-seq mapping, expression levels at the gene and isoform levels were determined and expression quantified using Cufflinks^47^ to generate FPKM estimates and HTseq^48^ to provide raw read counts. We used the R package DESeq2^49^ to determine the significance of differential expression in RNA-seq samples collected from the terminal ileum biopsies of 213 CD cases, 50 UC cases, and 35 controls of European descent from the RISK cohort.

### scRNA-seq data analyses

The details of library preparation and sequencing process have been described in a previously published work^35^. In total, we analyzed 70,226 cells from paired inflamed and uninflamed ileum from 11 CD patients. We aligned to the GRCh38 reference using the Cell Ranger v.2.1.0 Single-Cell Software Suite from 10X Genomics. The unfiltered raw matrices were then imported into R Studio as a Seurat object^50^. Genes expressed in fewer than three cells in a sample were excluded, as were cells that expressed fewer than 500 genes and with a UMI count less than 500 or greater than 60k. We normalized by dividing the UMI count per gene by the total UMI count in the corresponding cell and log-transforming. The Seurat integrated model was used to generate a combined CD model with cells from both inflamed and uninflamed samples retaining their group identity. We performed unsupervised clustering and differential gene expression analyses in the Seurat R package v.3.0.1. In particular, we used shared nearest neighbor graph-based clustering, in which the graph was constructed using from 1 to 30 principal components as determined by dataset variability shown in principal component analysis (PCA); the resolution parameter to determine the resulting number of clusters was also tuned accordingly. UMAP visualizations were produced using Seurat functions in conjunction with the ggplot2. Here, we extracted the average log expression of the 11 concerned genes across 31 annotated cell clusters.

### Ashkenazi Jewish sample identification

The Jewish HapMap dataset^6,51^ and 112 Europeans in the HapMap^52^ dataset were used to identify 100% Ashkenazi Jews among IBDGC samples. Jewish samples in Eastern Europe and the Middle East and Europeans were used as a reference panel to perform PCA, aiming to validate the distribution of genetically identified AJs compared to the AJ reference panel. Population structure analyses used 36 AJ references in Jewish HapMap datasets with all IBDGC candidates. PCA and population structure analysis were based on the same set of variants filtered by means of the following process: merging all IBDGC samples with all reference panels by Plink, then reducing linkage disequilibrium (LD) between markers (–indep-pairwise 50 5 0.2) by removing all markers with *r*^2^ > 0.2 (window size 50, step size 5)^53^, as well as markers in known high LD regions. Variants with MAF > 0.02^54^ and genotyping rate >95% across the dataset (excluding A/T, C/G mutations) and passing the above conditions were employed in PCA and STRUCTURE analyses. In population structure analyses, we removed Africans and Asians from IBDGC samples. Only ‘White’ samples, which include self-reporting AJ, self-reporting mixed AJs, and European were used as candidates (Supplementary Fig. 1). Accordingly, K was set to 2 to represent AJs, mixed-AJs, and Europeans to run fastSTRUCTURE^55^. The lowest AJ proportion (0.625) in the Ashkenazi reference panel was taken as AJ cutoff, and any IBDGC candidates passing this threshold were labeled as genetically identified AJs. To obtain a more precise cohort of AJ individuals, all genetically identified AJs were combined to perform a second round of population structure analysis compared to CEU and AJ reference panel using admixture where *k* = 2 gave the lowest cross-validation error and increased the lowest AJ fraction to 0.69 (Supplementary Fig. 9 and Supplementary Fig. 10). Therefore, 427 samples were removed from the dataset due to low AJ fraction. We validated the genetically identified AJs from fine-scale PCA plots without non-European populations. An independent AJ cluster can be seen from the validation PCA plots, which overlapped with the AJ reference panel (Supplementary Fig. 2). All genetically identified AJs were plotted as sky-blue points in PCA plots. Another PCA compared all of the genetically identified AJs without any reference panels. Samples were color coded based on either source of dataset or case-control status, indicating that there are no confounding factors due to data sources or phenotypes (Supplementary Fig. 11). We included the first 10 principal components as covariates in the variant- and gene-level association tests to overcome the impact of any remaining population stratification on the results.

### Variant annotation

Multiallelic sites were split into single variants using bcftools before annotation. Variant Effect Predictor (VEP, v90)^56^ and SnpEff (v4.2)^57^ were employed for annotation. We used a Python script to manage a parallel running of two annotation methods and merging of results at variant level by removing redundant annotation results. CADD scores (v1.3) were added into the final results. All annotation processes were conducted based on GRCh37 genome coordinates.

### High impact rare variant filtration

We retained rare and high-impact genetic variants using the following criteria: (1) Maintained variants with DP > 10, MQ > 40 in VCF file to control the base quality. (2) Utilized Variant Effect Predictor (VEP) to determine the effect of all variants. Variants were filtered by ‘consequence’ of VEP annotations, high impact variants were retained by virtue of their impact on genome functions: ‘missense variants’, ‘start lost’, ‘stop lost’, ‘stop gained’, ‘splice_acceptor_variant’, ‘splice_donor_variant’, ‘inframe_insertion’, ‘inframe_deletion’, ‘protein_altering_variant’, ‘start_retained_variant’, ‘stop_retained_variant’ and ‘frameshift_variant’. (3) Removed variants with MAF > 0.01 according to gnomAD AJ allele frequency. When gnomAD AJ allele frequency was missing for a given variant, we used its allele frequency from our AJ cohort. (4) Employed Mutation Significance Cutoff (MSC)^58^ to control the false-negative rate of predicted deleterious mutations by well-established predictors, like CADD, SIFT, and Polyphen-2. Here we retained all variants with CADD^59^ scores larger than the lower boundary of the 95% confidence interval of the corresponding gene’s pathogenic mutation’s CADD score. (5) Genes that are highly mutated in healthy individuals are unlikely to be disease-causing. Therefore, an estimate of accumulated mutational damage of each human gene can be particularly helpful in filtering out genes that are irrelevant to disease or phenotype. The Gene damage index (GDI)^60^ is an indicator of genes that are highly polymorphic in the general healthy population, and hence are unlikely to be disease-causing. Only variants in genes with a GDI < 13.34 (the cutoff proposed for human diseases under the generalized model) were retained for further analyses. (6) Variants frequent in a given exome cohort, but absent or rare in public databases, have also been reported and treated as non-pathogenic variants (NPV)^61^. We removed all variants that were described in the precalculated ‘blacklist’. The remaining variants were used for further analyses.

### Association analyses

We performed SKAT-O analyses on aggregations of high-impact variants to test associations between genes and IBD (CD/UC) disease status. We filtered variants using gnomAD AJ MAF < 0.01. However, a few sites exceeded a MAF of 0.01 among our AJ cohort, which added extra power to their corresponding genes in the SKAT association tests. Therefore, we conservatively filtered out these variants from SKAT-O analysis by adding a ‘maf <0.01’ parameter in SKAT functions. A model-based association test was conducted by Plink in validating pathway-derived high-impact variants; odds ratio and *P*-values were obtained from logistic regression running in Plink1.90. We performed a collapsing analysis that considered only synonymous variation as a neutral model to estimate the degree of inflation due to population substructure or possible technical artifact. We also performed a collapsing analysis of controls-*vs*.-controls using high-impact rare variants to check the possible heterogeneities in controls between two datasets. Neither analysis indicated a significant level of inflation in the results (Supplementary Figs. 12 and 13).

### Meta-analyses

Meta-analyses were conducted using Raremetal^62^ to validate the SKAT-O gene burden significant genes (*P* value <0.01). Significant genes in Raremetal analyses were compared with SKAT analyses to check which genes were replicated in meta-analyses. The IBDGC exomes were divided into two datasets according to the sources of recruitment. Dataset 1 comprised 1058 IBD cases and 436 unaffected controls, whilst dataset 2 comprised 676 IBD cases and 2283 unaffected controls. Raremetalworker was used to calculate a single variants’ statistical summary for our datasets 1 and 2, respectively. As with the SKAT-O analysis, only high-impact variants in each dataset were investigated. Next, we ran Raremetal to collect statistical results on independent datasets from Raremetalworker with SKAT meta function. The aggregations of high-impact variants were supplied in this process to perform gene level metal analyses. SKAT function was used as the association test method in running Raremetal.

### Phenome-wide association analysis

To evaluate the potential pleiotropic effects of the candidate genes from SKAT-O analysis and pathway analyses, we performed gene-level PheWAS using the whole exome sequencing dataset and diagnostic information from 30,845 patients from the Mount Sinai BioMe Biobank. The cases were collected according to the ICD-10-CM codes, phenotypes having at least 100 cases being kept for PheWAS. To minimize the bias from controls which could include similar or relevant phenotypes with cases, we collected samples with ‘Z00.00’ (Encounter for general adult medical examination without abnormal findings) as a pool of controls. Meanwhile, given that the number of cases was relatively small compared to the control set, to minimize an inflation due to the extremely unbalanced numbers of cases versus controls, for each phenotype we randomly selected a subset from the pool of controls to keep the ratio of cases to controls as 1:10. The overlapping individuals were removed from the controls set. A principal component analysis (PCA) was applied to all BioMe 30,845 exomes prior to the PheWAS analyses, with the first two components being used to adjust the population structure in the association tests. An R script was written to conduct the gene-level PheWAS by combining the ‘PheWAS’ package and the ‘SKAT’ package. For each candidate gene, the high impact rare variants from the Ashkenazi Jewish IBD cohort were used to repeat SKAT-O tests to identify associations with each BioMe phenotype. To assess whether the signals of collapsed rare variants in PheWAS were independent of the nearby common variant association signals, we performed conditional SKAT-O for the significant associations (Supplementary Results, Supplementary Data 18).

### Polygenic risk score and machine-learning prediction

Polygenic risk scores (PRS) generate quantitative metrics of individuals based on the cumulative effects of risk alleles. It can simply be a summation of the number of risk alleles across associated genes or an accumulation of risk variants weighted by effect size. Traditionally, genome-wide significant sites have been employed to generate PRS. Here, we aimed to keep all selected variants in PRS derivation. We calculated PRS for each individual based on selected high-impact variants by using the LDpred algorithm^63^. Unlike variant pruning approaches, LDpred infers the posterior mean effect size of each variant by using a prior on effect sizes and LD information from an external reference panel. We used three different external panels to compare the consequences for classification models; one was the AJ IBD GWAS summary from Hui et al.^38^, the second was the European IBD GWAS summary from Liu’s study^2^, the third was the GWAS statistics on half of our IBD cases and controls; the remaining set of IBD cases and controls was used as a validation dataset. Variants with ambiguous strands (A/T, C/G) were removed from all high-impact sites in the validation dataset. There were seven PRS generated because of the fraction *p* of non-zero effects in the prior (1, 0.3, 0.1, 0.03, 0.01, 0.003, 0.001). All seven PRS were used as features in a Random Forest classification model, whilst the average AUC of 10-fold cross-validation was used to compare prediction performance.

We then applied a multi-layer feedforward artificial neural network, also known as convolutional neural network (CNN), to build the prediction model using all the rare high-impact variants as described above. Grid search was performed to determine the best parameter settings including numbers of hidden layers, number of neurons in each layer, activation functions of the layers, dropout ratio as well as parameters for L1 and L2 regularization. 10-fold cross-validation was performed to estimate the AUC of the tuned model (7-layer CNN model with dropout ratio of 0.19, L1 of 0.002, and L2 of 0.009).

### Pathway and enrichment analyses

We used several independent pathway and enrichment methods to obtain an IBD candidate gene list using known IBD genes as a reference. The final highly credible gene list was finalized by extracting intersection genes across IBD gene sets resulting from each pathway and enrichment analyses. The candidate genes were defined as genes which passed the relaxed threshold *P* < 0.01 in a SKAT-O IBD case-control study. 127 genes were obtained from IBD-specific SKAT-O case-control association studies. The IBD known genes were collected from studies summarizing IBD, CD, and UC genes and fine mapping efforts of identified IBD loci harboring associations mapped to single variants with greater than 95% certainty^3,4^, which comprised a list of 157 IBD-associated genes (Supplementary Data 5).

#### Ingenuity pathway analysis

First, we ran ingenuity pathway analysis (IPA) on the IBD candidate genes^64^, where ‘Cancer’, ‘Organismal Injury and Abnormalities’ and ‘Gastrointestinal Disease’ ranked as the top three most correlated diseases for the input genes. To select genes most relevant to IBD, we used the ‘Gastrointestinal Disease’ panel to extract genes belonging to its sub-phenotypes: ‘inflammation of gastrointestinal tract’, ‘inflammation of small intestine’, and ‘colitis’. We repeated the process on CD-specific and UC-specific genes.

#### ToppGene

We used ToppGene to select candidate genes from SKAT-O significant genes^65^. ToppGene can prioritize candidate genes based on functional similarity to a training gene list. Here we used known IBD genes for training; therefore, all IBD candidate genes were ranked by training model. Each gene was assigned scores and *P* values representing functional similarities with known genes in relation to GO terms, disease phenotypes, pathways, etc. We retained those genes with *P* values <0.05 from candidate genes as the IBD gene list.

#### GIANT

Gene function module contains clusters of genes which have similar biological functions or a shorter biological distance with each other. We used GIANT function to obtain function modules from our candidate genes^66^. GIANT applies community detection to find cohesive gene clusters from a provided gene list and a selected relevant tissue. The most IBD-relevant module was selected according to its immunological function with global tissue condition. The non-redundant genes within these identified function modules form the IBD gene list from GIANT.

#### The human gene connectome (HGC)

The HGC is the set of all biologically plausible routes, distances, and degrees of separation between all pairs of human genes^11^. A gene-specific connectome contains the set of all available human genes sorted on the basis of their predicted biological proximity to the specific gene of interest. Here, the known IBD genes are the genes of interest; for each known IBD gene, we calculated the distances to every other known IBD gene *D*_*ij*_, assuming that we have a SKAT-O significant gene set A $A=\left\{{a}_{1},{a}_{2},\ldots,{a}_{m}\right\}$ and IBD known gene set B $B=\left\{{b}_{1},{b}_{2},\,\ldots,\,{b}_{n}\right\}$, the biological distance derived from HGC between genes from two gene sets is be represented as

1 $${D}_{{ij}}={{{{{{\rm{Distance}}}}}}}\left({a}_{i},\,{b}_{j}\right)$$

For each candidate gene ${a}_{i}$ in set A, its average distance to all known IBD genes was denoted as:

2 $${D}_{{{{{{{\rm{candidate}}}}}}}}={D}_{i}^{A}={{avg}\atop{j\!\in \left\{1,\,\ldots,n\right\}}}\left({D}_{{ij}}\right)$$

Then the overall average distance between A and B was used to represent the biological distance of candidate gene set to the known IBD gene set B:

3 $${{avg}\atop{i,\, j}}\left({D}_{{ij}}\right)={{avg}\atop{i\!\in \left\{1,\,\ldots,m\right\}}}\left({{avg}\atop{j\!\in \left\{1,\,\ldots,n\right\}}}\left({D}_{{ij}}\right)\right)$$

Therefore, for the known IBD gene set, we calculated the average distance within IBD genes (*D*_*IBD*_) by checking the overall average distance from gene set B to itself. For each candidate gene, if its *D*_candidate_ was shorter than *D*_*IBD*_, it was retained as a plausible IBD gene to contribute to the HGC IBD gene list.

At the gene set level, randomly resampling tests were conducted to demonstrate that SKAT significant genes having a shorter average biological distance to known IBD genes than random genes was not due to chance alone. For each resampling iteration, a set of genes having equal size with SKAT-O significant genes was randomly sampled from the gene pool (all genes in SKAT-O inputs) and the average distances of random sets (*D*_random_) were calculated following Eq. (3). Similarly, the distance of SKAT-O significant genes was obtained (*D*_*SKAT*_) as a cutoff. The resampling tests were conducted for 1000, 5000, and 10000 iterations, the *P-*value representing the number of iterations in which random sets had a shorter biological distance compared to the SKAT-O significant gene set (*D*_random_ < *D*_*SKAT*_) among all iterations in each resampling process.

### Gene prioritization based on pathway analyses results

The SKAT-O significant IBD genes (*P* < 0.01) were prioritized by their biological importance in IBD pathways. One gene may be involved in multiple pathways or IBD gene function modules where other IBD genes also exist. Biological importance was measured by counting the total number of IBD known genes in significant pathways or function modules, resulting from each enrichment analysis. The biological importance scores were added up as the final score to prioritize genes. Specifically, we collected gene sets for pathways and gene function modules from the following pathway/function analyses: (1) InnateDB^67^ pathway analysis: pathways with *P* value <0.05. (2) InnateDB gene ontology analysis: gene list with *P* value <0.05. (3) Networkanalyst^68^: first degree genes to each candidate genes were interrogated for counting IBD known genes. (4) IPA: canonical pathways with *P* value <0.01 were employed. (5) Human Gene Connectome: *P* values <0.01 for biological distance were collected in order to calculate the number of IBD genes.

### Ethics statement

In all cases, informed consent was obtained using protocols approved by the local institutional review board in all participating institutions. All patients and controls gave informed consent, and the study was approved by the ethics review committees of each participating hospital. Informed consent was obtained using protocols approved by each local institutional review board.

### Reporting summary

Further information on research design is available in the Nature Portfolio Reporting Summary linked to this article.

## Supplementary information

Supplementary Information

Peer Review File

Description of Additional Supplementary Files

Supplementary Data 1-18

Reporting Summary

## Data Availability

The single-cell RNA-seq raw data used in this study are available at the NCBI GEO database under accession code GSE134809. The RNA data used in this study is available in dbGaP Study Accession: phs001642.v1.p1 (https://www.ncbi.nlm.nih.gov/projects/gap/cgi-bin/study.cgi?study_id=phs001642.v1.p1). The DNA data used in this study is available in Gene Expression Omnibus (GEO) Series GSE57945. The Mutation Significance Cutoff database for selecting high-impact variants is available at https://lab.rockefeller.edu/casanova/MSC. The gene- and variant-level association data generated in this study are provided in the supplementary data. The raw sequencing data and raw bulk RNA-seq data are protected and are not available due to data privacy laws, which can be available based on reasonable request to IBDGC consortium (https://www.ibdgc.org/).

## Code availability

The python code for prioritizing candidate genes can be found at https://lab.rockefeller.edu/casanova/HGC. The in-house script for running gene level PheWAS can be found at https://gitlab.com/wym0072003/multi-phewas. Standard software guidelines were followed for other scripts used in this study.
