# Supplementary material for: Identifying high-impact variants and genes in exomes of Ashkenazi Jewish inflammatory bowel disease patients
Source: Nat Commun. 2023 Apr 20;14:2256. doi: 10.1038/s41467-023-37849-3 (PMC10119186; doi:10.1038/s41467-023-37849-3)
Supplement: Supplementary file 1 — Supplementary Information [file 41467_2023_37849_MOESM1_ESM.pdf]

Supplementary Materials for

Identifying high-impact variants and genes in exomes of Ashkenazi Jewish inflammatory bowel disease patients

Yiming Wu, Kyle Gettler, Meltem Ece Kars, Mamta Giri, Dalin Li, Cigdem Sevim Bayrak, Peng Zhang, Aayushee Jain, Patrick Maffucci, Ksenija Sabic, Tielman Van Vleck, Girish Nadkarni, Lee A. Denson, Harry Ostrer, Adam P. Levine, Elena R. Schiff, Anthony W. Segal, Subra Kugathasan, Peter D. Stenson, David N. Cooper, L. Philip Schumm, Scott Snapper, Mark J. Daly, Talin Haritunians, Richard H. Duerr, Mark S. Silverberg, John D. Rioux, Steven R. Brant, Dermot McGovern, Judy H. Cho, Yuval Itan

Correspondence: yuval.itan@mssm.edu

This PDF file includes:

Supplementary Results
Supplementary Figs. 1 to 13
Supplementary Data 1 to 18

Supplementary Results

Identifying IBD candidates genes using pathway analysis and functional module analysis

We used IPA to identify candidate gene-related pathways, diseases and biological functions. The 'Gastrointestinal Disease' term ($P = 1.06\times10^{-2} – 6.22\times10^{-10}$) was ranked 2nd among the 'Top Disease and Biological Functions' categories (the top term being 'Cancer' whilst the 3rd term was 'Organismal Injury and Abnormalities'). Of the 34 sub-functions of 'Gastrointestinal Disease', we collated 104 genes from the most significant function module ($P = 6.22\times10^{-10}$) (Supplementary Data 6). We employed ToppGene to rank candidate genes according to their relatedness to known IBD genes based on functional annotation and protein interaction network. The candidate genes were sorted by statistics generated by integrating functional annotations and protein interaction networks. 43 genes with $P < 0.05$ were selected as the top candidates (Supplementary Data 6). We used the module detection tool GIANT to identify IBD-related genes from our candidate genes. Using GIANT's default setting (global tissue), we identified five functional modules, one of which was highly correlated with immunological functions including macrophage activation, cell activation and leukocyte activation, from which we extracted its 22 genes (Supplementary Data 6). We employed HGC to calculate the average distance of each candidate gene to known IBD genes, then obtained 24 genes with an average HGC distance lower than 11.13 (the cutoff based on the average distance between known IBD-associated genes) for further analyses (Supplementary Data 6).

Gene-level PheWAS using ~30K whole exomes and phenotypic information from Mount Sinai Hospital's BioMe BioBank

Gene-level PheWAS were performed for the 11 candidate genes. In total, 1,703 phenotypes with at least 100 cases exhibited a phenome-wide significance level of 2.9×10^{-5}. The association between Parkinson's disease and *LRRK2* was above the phenome-wide level of significance (G20, $P = 7.36\times10^{-12}$). Previous analyses have demonstrated that *LRRK2* can play important roles in both PD and IBD[1]. Here, the same set of high impact rare variants has been used for the analysis of both phenotypes; therefore, the results obtained may indicate that the comorbidity of PD and IBD is driven by *LRRK2* rare variants. Thus, *NOD2* was the most relevant gene to IBD, being significantly associated with Crohn's disease of the small intestine (K50.00, $P = 2.63\times10^{-5}$) and Crohn's disease (K50.90, $P = 1.73\times10^{-3}$). Other than *NOD2*, the *ICAM1* gene was found to be associated with ulcerative (chronic) pancolitis without complication (K51.00, $P = 2.65\times10^{-2}$) in BioMe. In addition to IBD, *ICAM1* is associated with type 1 diabetes mellitus without complications (E10.9, $P = 9.09\times10^{-4}$). It was already known that type 1 diabetes patients have a higher risk of developing inflammatory bowel disease[2,3], and that the *ICAM1* gene is potentially associated with the comorbidity of both diseases. The other candidate genes did not display significant associations with IBD in the BioMe Biobank PheWAS analyses. This is probably because of the small number of high impact rare variants being covered in the tested exomes combined and because of the limited IBD sample size (678 IBD samples, including CD and UC) in BioMe. We further performed conditional analysis to evaluate whether the signals of collapsed rare variants in PheWAS were independent of the nearby common variant association signals (±100 Kbp up- and down-stream) following previous study[4]. To identify most significant nearby variants, we first extracted all common variants in the vicinity (within 100Kbp) of significant rare variants ($p < 0.05$) from imputed array data of the Mount Sinai BioMe BioBank, then performed single variant tests with SKAT-O using the same parameters as the gene level test (Method). All associations remained significant after the conditional analysis (Supplementary Data 18). Because *TYK2* is a known type 1 diabetes-associated gene which is located in the proximity of *ICAM1*, we also performed a conditional analysis for the most significant common variant of *TYK2* located in ±100 Kbp up- and down-stream of *ICAM1*. The results showed that the *ICAM1*-type 1 diabetes mellitus association was still signficant after conditional testing (Supplementary Data 18).

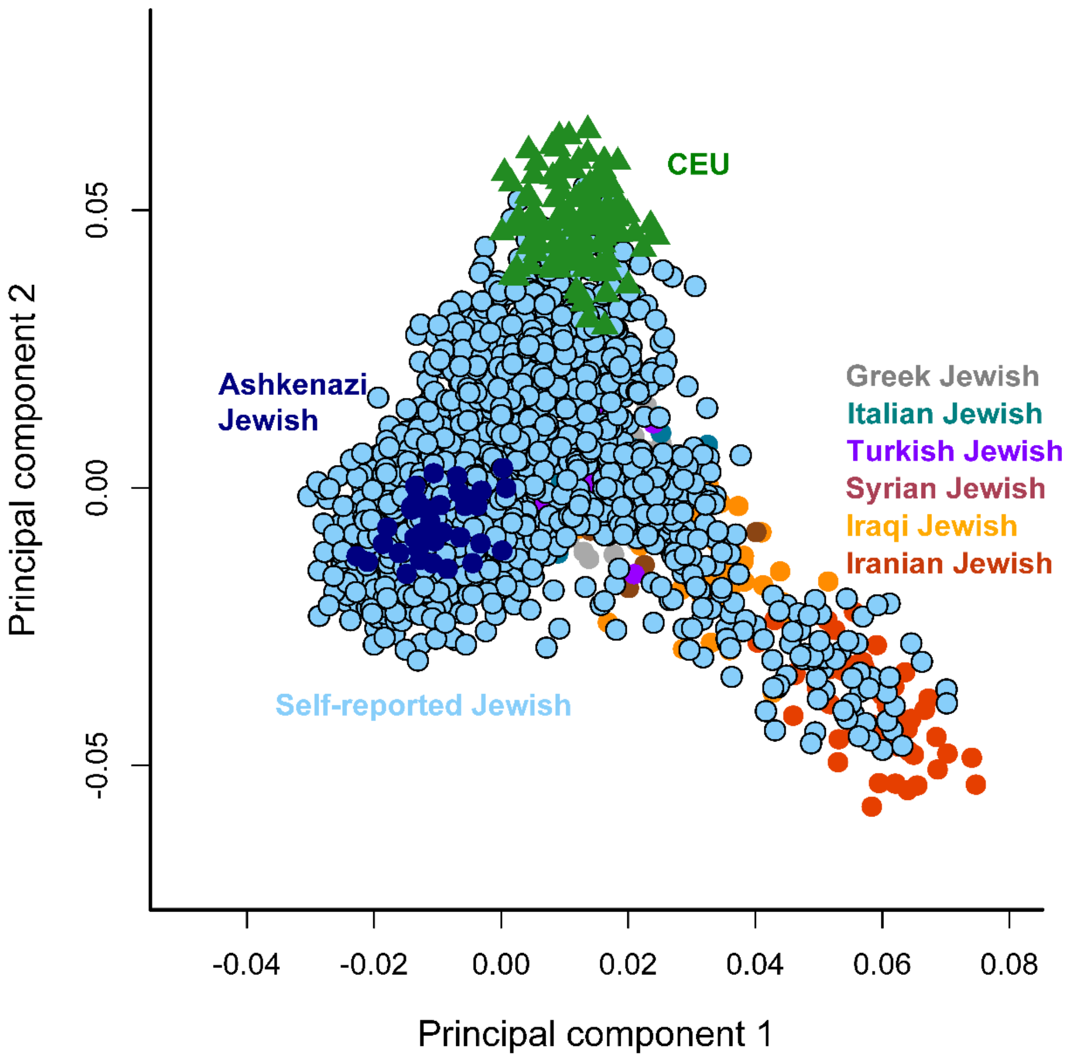

Supplementary Fig. 1.

Non-European samples were removed from all participants to perform a fine-scale PCA on general Europeans. The PCA results indicated that 2,767 self-reported Jewish samples (sky-blue dots) included both European and mixed-Jewish samples.

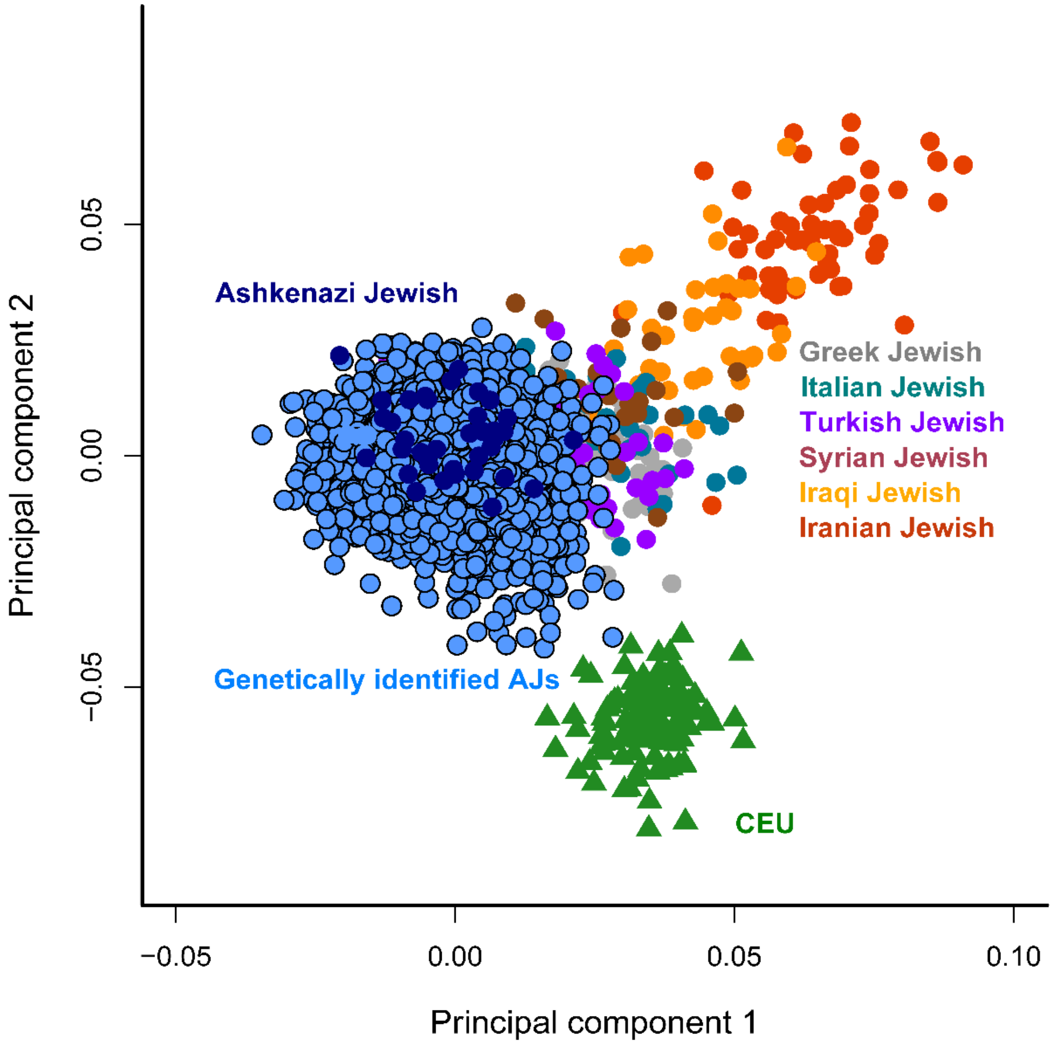

Supplementary Fig. 2.

Genetically identified AJ samples from IBDGC dataset 2

As with dataset 1 (Fig 1.b), the genetically identified AJs (5,254 samples including cases other than IBD) form an independent cluster, which overlaps with the AJ reference panel while exhibiting some distance to the European reference panel.

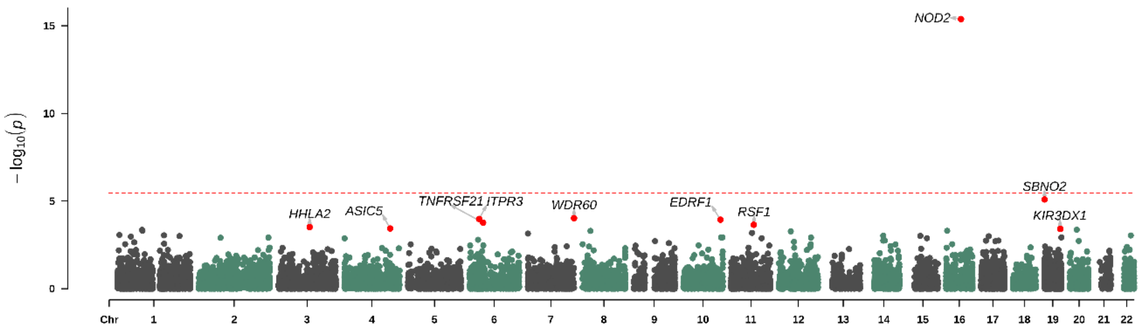

Supplementary Fig. 3.

A Manhattan plot for Crohn's disease-specific analysis

A gene level Manhattan plot showing significantly associated genes that passed the Bonferroni adjusted threshold in the Crohn's disease-specific SKAT-O test. Compared to the IBD-specific Manhattan plot (Fig 2.c), only *NOD2*, a well-known CD-specific gene, remained significant at the Bonferroni adjusted *P* value threshold. All dots represent negative log unadjusted *P* values.

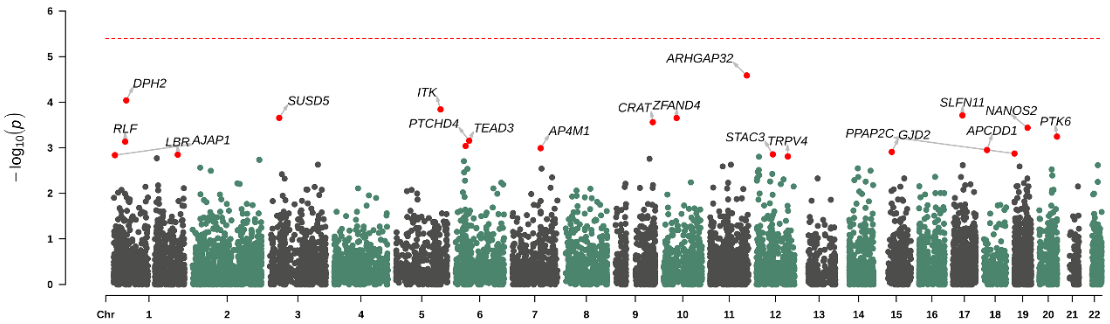

Supplementary Fig. 4.
A Manhattan plot for ulcerative colitis-specific analysis
A gene level Manhattan plot showing significantly associated genes that passed the Bonferroni adjusted threshold from the UC-specific SKAT-O test. In contrast to the CD-specific test, the *NOD2* signal lost its significance. However, we did not observe genes associated with UC, probably because of small sample size (458) of UC cases. All dots represent negative log unadjusted *P* values.

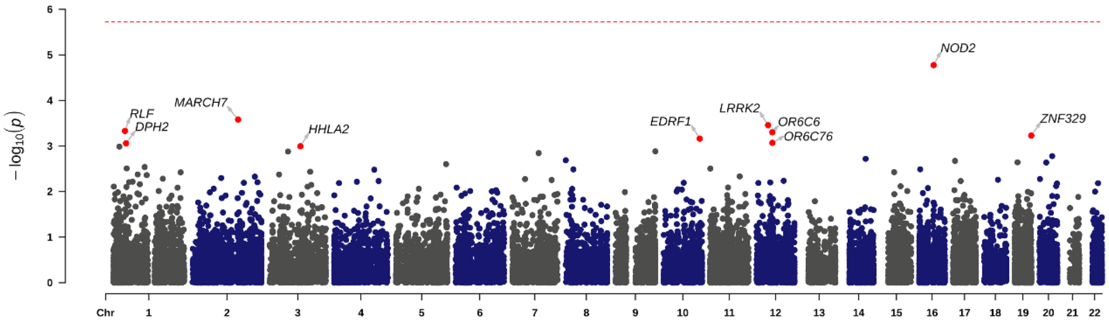

Supplementary Fig. 5.
A Manhattan plot for variant level associations from IBD analysis
Variant level association test on IBD cases and controls, each point representing a variant in association analysis using logistic regression; the genes harboring the variants are labeled in this Manhattan plot. Cross-checking with gene level results (Fig 2. a), the top genes generally harbor one or more variants. However, no variant passed the genome-wide association threshold ($P = 5\times10^{-8}$) in the variant level association test. All dots represent negative log unadjusted P values, the statistical test is two-sided.

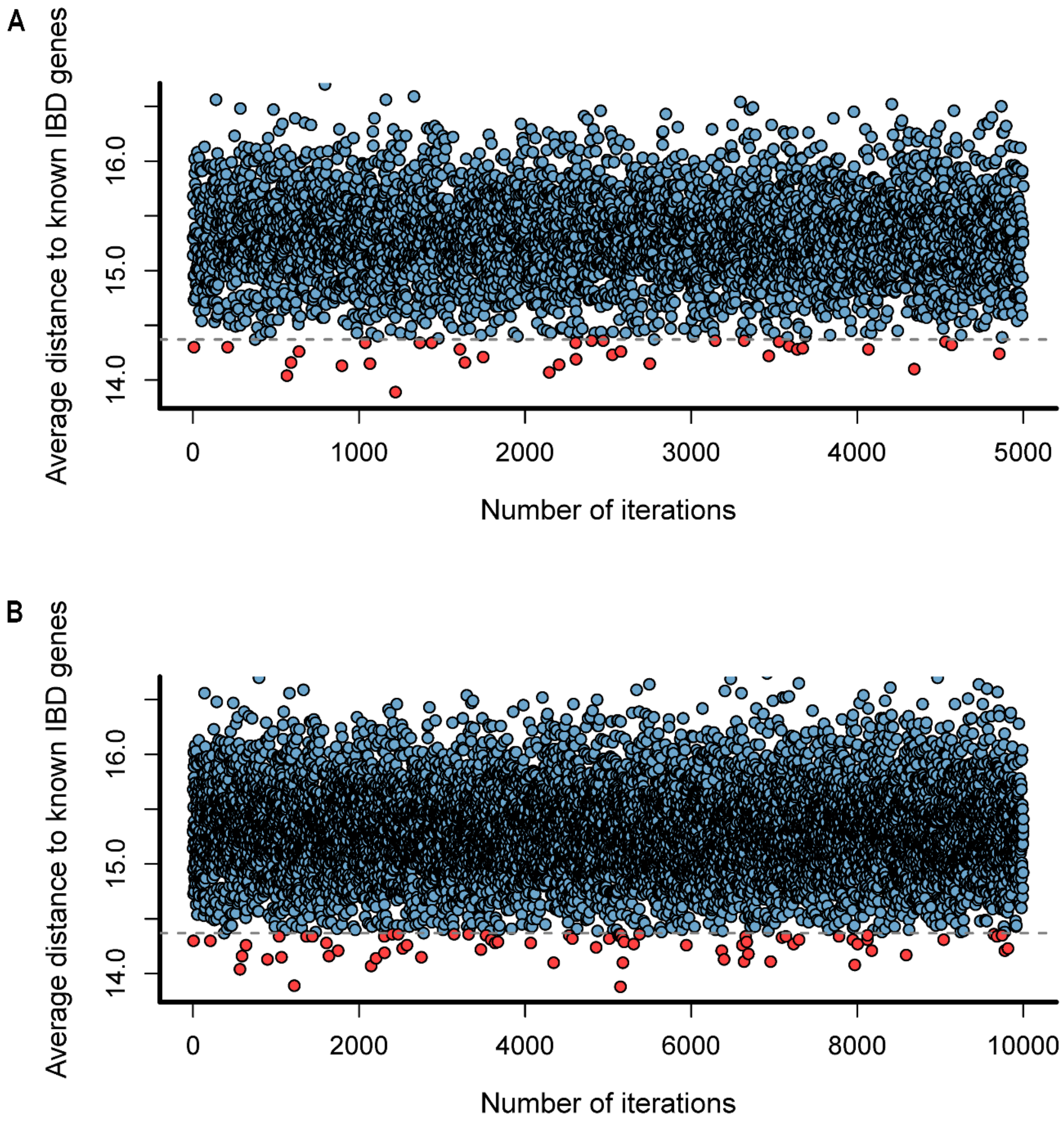

Supplementary Fig. 6.
Average biological distances from random gene sets to known IBD genes.
Gene sets comprising 127 random genes were resampled in each iteration (the same size as IBD-associated genes with $P < 0.01$) to calculate the average biological distance to known IBD genes. The calculated average distance was compared to that of IBD-associated genes to the known IBD genes (14.37). The *P* value denotes the number of iterations with distance lower than the cutoff results from IBD-associated genes. (**A**), the resampling iteration was 5,000, $P = 0.007$. (**B**), the resampling iteration was 10,000, $P = 0.0072$. All *P* values are unadjusted, the statistical tests are two-sided.

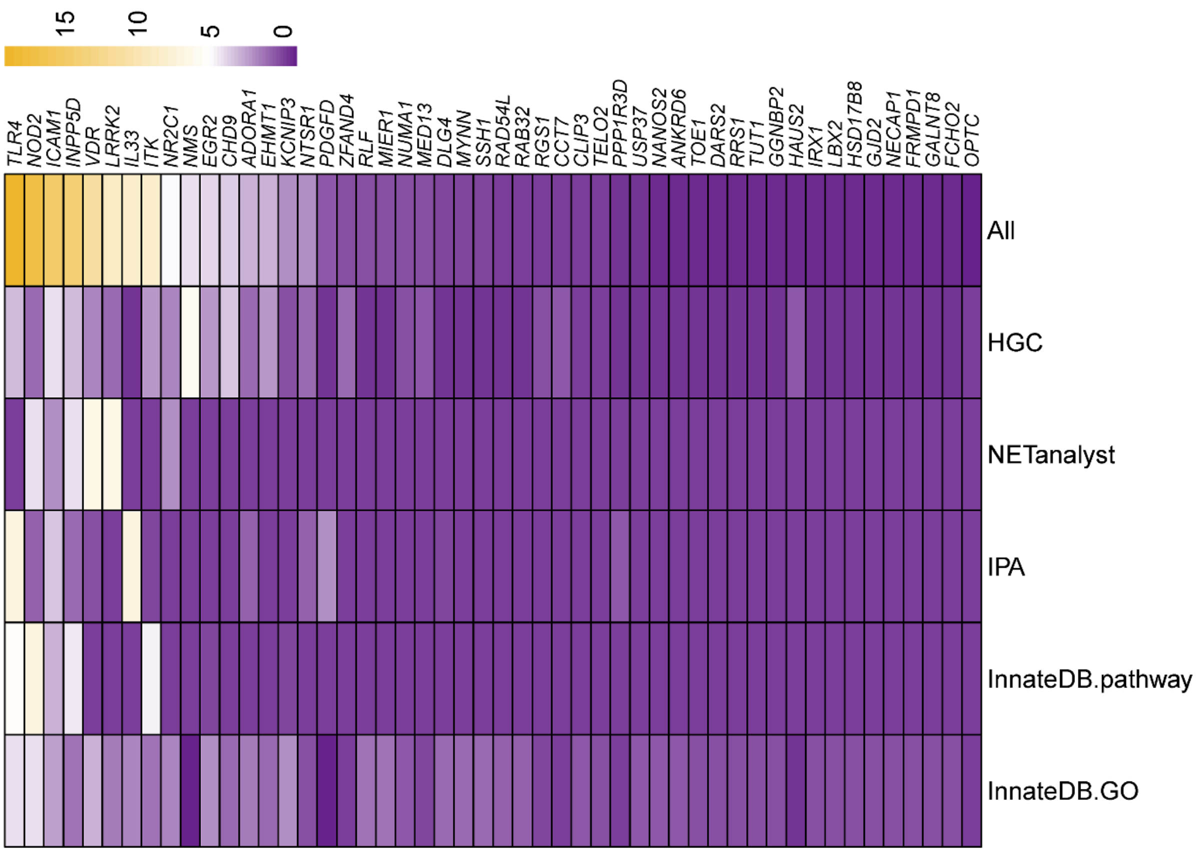

Supplementary Fig. 7.

A heatmap summarizing gene prioritization results from pathway and function analyses

Gene prioritization results, pathway and gene function module were obtained from pathway and function analyses. Genes were weighted according to their correlation or the number of shared pathways with known IBD genes (Online Methods). Each heatmap square represents a prioritization score, a higher score denoting a higher probability of sharing pathways and function modules with known IBD genes. The original matrix was scaled to 0-18 after normalization for displaying results in good contrast; only the top 50 genes are shown in the heatmap.

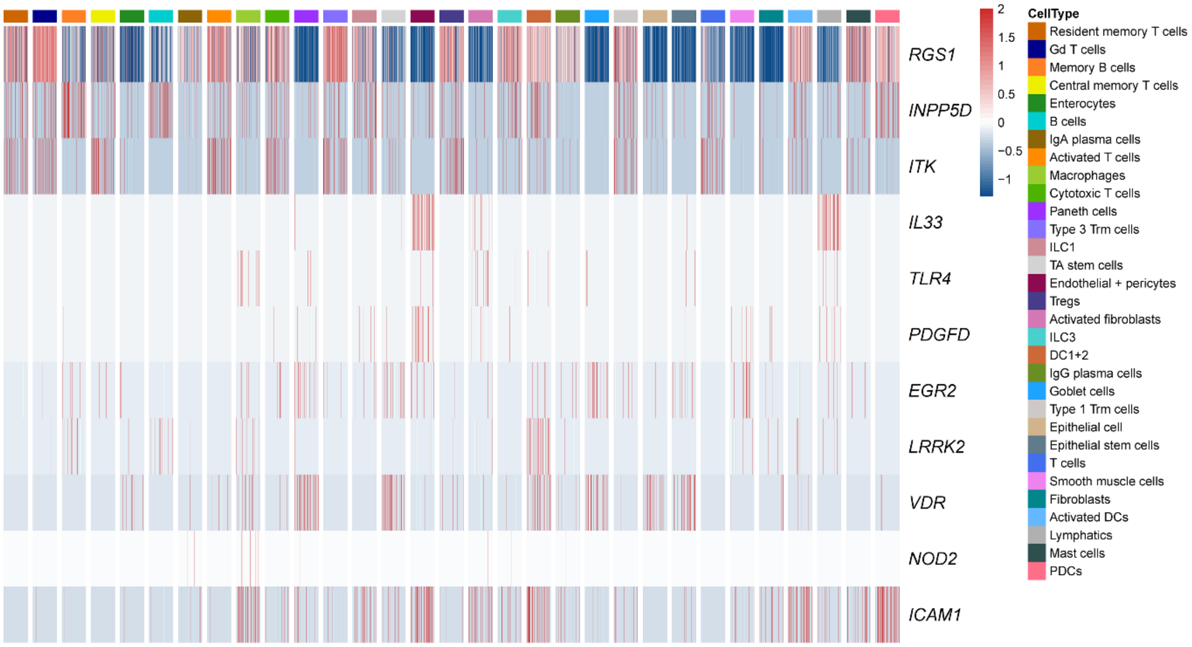

Supplementary Fig. 8.
Expression of the 11 candidate genes in different cell types
Heatmap showing the expression levels of the 11 candidate genes in cells across the 31 cell type clusters. The expression levels in cells were scaled by genes.

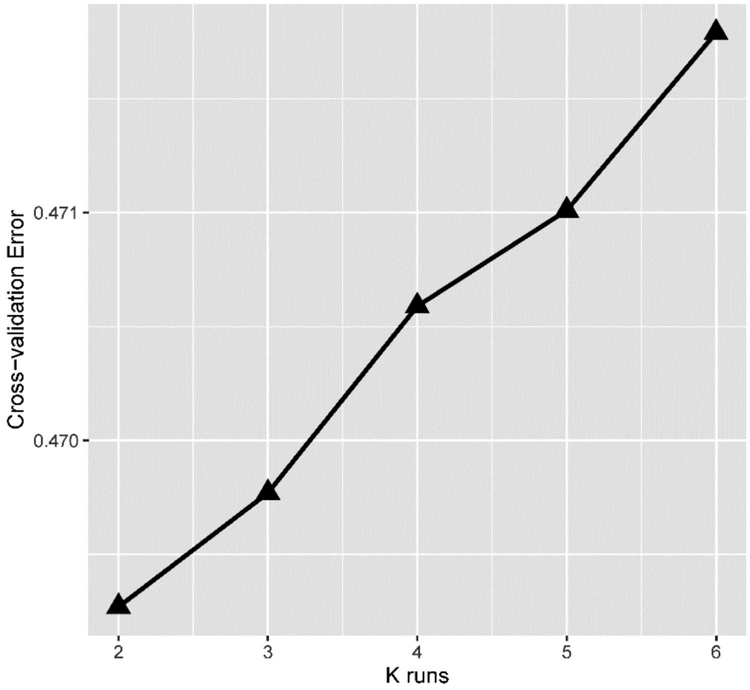

Supplementary Fig. 9

Cross-validation procedure implemented by Admixture to choose the best *K*.

Cross-validation errors for the identifications of AJ samples were obtained by comparing all AJ candidates to the AJ reference panel and the European panel. $k = 2$ resulted in the lowest cross-validation error.

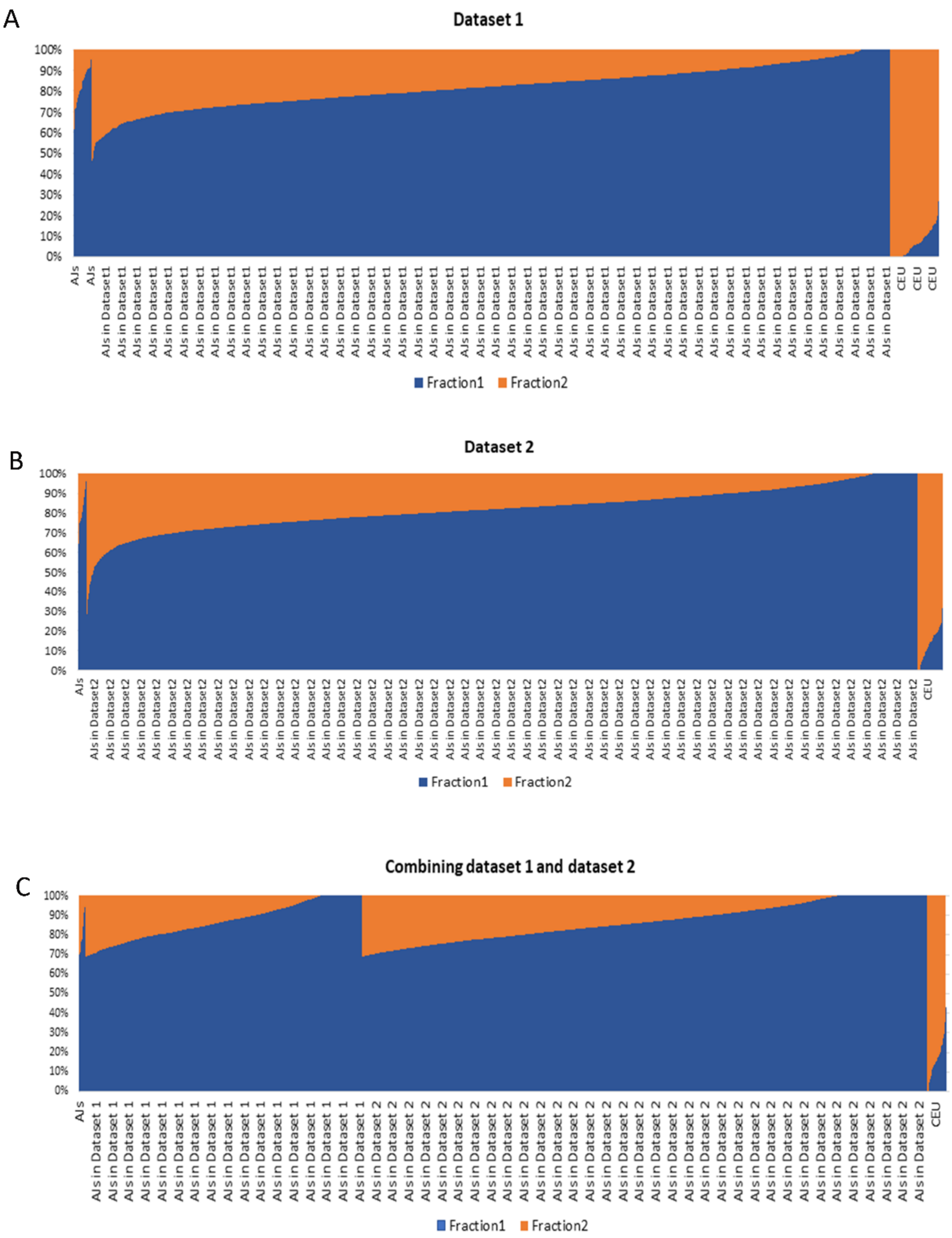

Supplementary Fig. 10.
Admixture analyses on genetically identified AJs.
The admixture analyses for the A) Dataset 1, B) Dataset 2, and C) combined dataset together with the samples from the AJ reference panel and the CEU population from the 1000 Genomes Project after further filtering samples with a relatively low AJ fraction (AJ fraction < 0.69, the lowest AJ fraction in the AJ reference panel).

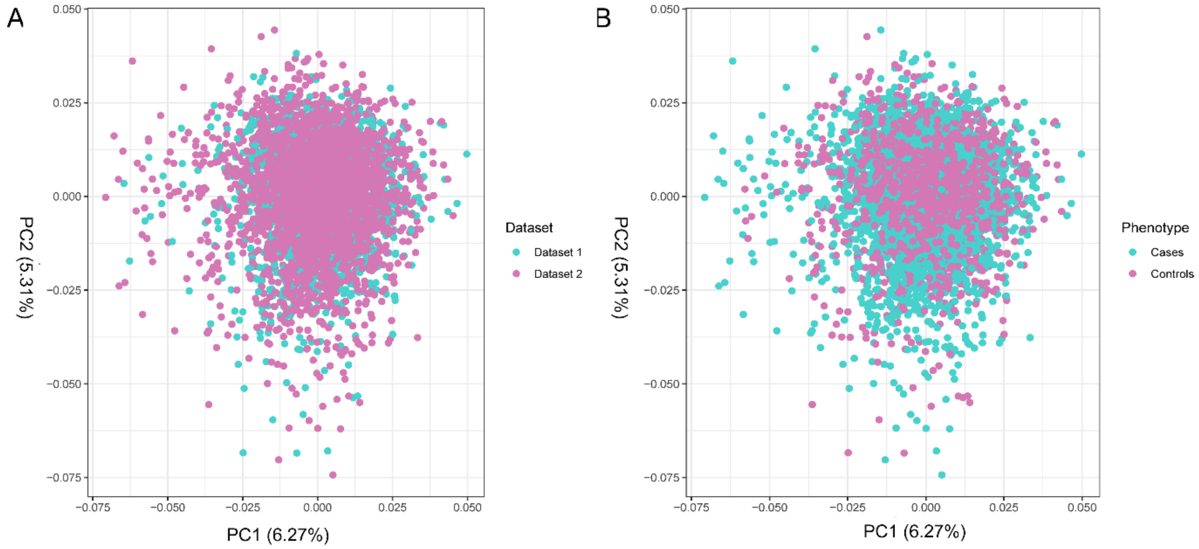

Supplementary Fig. 11.
Principal component analysis of genetically identified Ashkenazi Jewish samples (without AJ references).
Individuals are color coded based on either source of dataset (A) or case control status (B).

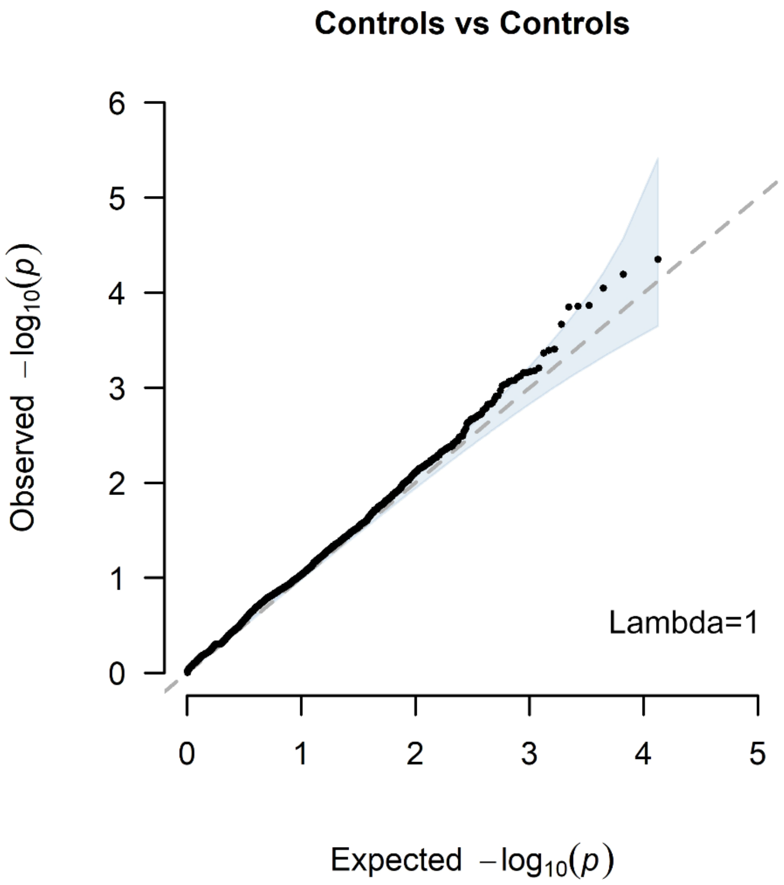

Supplementary Fig. 12.
Q-Q plot with 95% confidence bands for SKAT-O analysis of controls *vs.* controls by using only controls from 2 AJ-IBD datasets. All *P* values are unadjusted, the statistical test is two-sided.

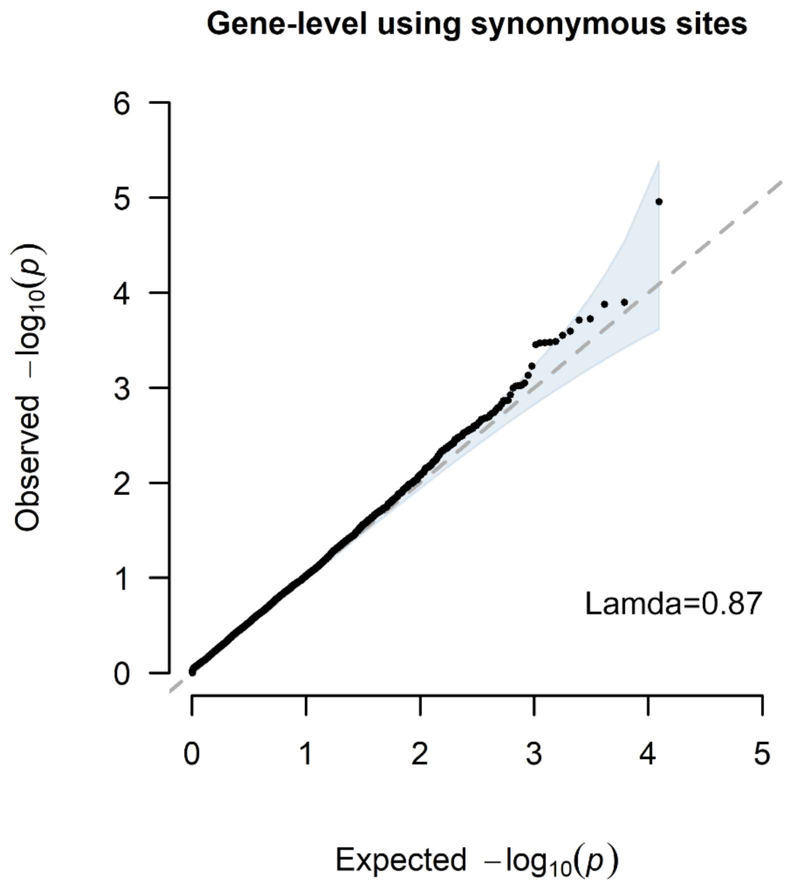

Supplementary Fig. 13.
Q-Q plot with 95% confidence bands for collapsing analysis of synonymous variants for IBD cases *vs.* controls. All *P* values are unadjusted, the statistical test is two-sided.

Supplementary References

1. Hui, K.Y., *et al.* Functional variants in the *LRRK2* gene confer shared effects on risk for Crohn's disease and Parkinson's disease. *Sci. Transl. Med.* **10**, eaai7795 (2018).
2. Kang, E.A., *et al.* Increased risk of diabetes in inflammatory bowel disease patients: a nationwide population-based study in Korea. *Journal of Clinical Medicine* **8**, 343 (2019).
3. Kurppa, K., *et al*. Coeliac disease in children with type 1 diabetes. *Lancet Child Adolesc Health* **2**, 133-143 (2018).
4. Zhao, Z., *et al*. UK Biobank whole-exome sequence binary phenome analysis with robust region-based rare-variant test. *Am. J. Hum. Genet.* **106,** 3-12 (2020).
