# Supplementary material for: Identifying high-impact variants and genes in exomes of Ashkenazi Jewish inflammatory bowel disease patients
Source: Nat Commun. 2023 Apr 20;14:2256. doi: 10.1038/s41467-023-37849-3 (PMC10119186; doi:10.1038/s41467-023-37849-3)
Supplement: Supplementary file 3 — Description of Additional Supplementary Files [file 41467_2023_37849_MOESM3_ESM.pdf]

Description of Additional Supplementary Files

File Name: Supplementary Data 1
Description: IBD-associated genes from SKAT-O analysis on AJ IBD cohort using high impact rare variants. SetID, gene IDs tested by SKAT-O analyses. P.value, P values of SKAT. M, the number of individuals with minor alleles. N.Marker.All, the number of SNPs in the genotype matrix. N.Marker.Test, the number of SNPs used for the test. MAP, minimum possible P values. Method.bin, type of method to compute a P value.

File Name: Supplementary Data 2
Description: CD-associated genes from SKAT-O analysis on AJ CD cohort using high impact rare variants. SetID, gene IDs tested by SKAT-O analyses. P.value, P values of SKAT. M, the number of individuals with minor alleles. N.Marker.All, the number of SNPs in the genotype matrix. N.Marker.Test, the number of SNPs used for the test. MAP, minimum possible P values. Method.bin, type of method to compute a P value.

File Name: Supplementary Data 3
Description: UC-associated genes from SKAT-O analysis on AJ UC cohort using high impact rare variants. SetID, gene IDs tested by SKAT-O analyses. P.value, P values of SKAT. M, the number of individuals with minor alleles. N.Marker.All, the number of SNPs in the genotype matrix. N.Marker.Test, the number of SNPs used for the test. MAP, minimum possible P values. Method.bin, type of method to compute a P value.

File Name: Supplementary Data 4
Description: Association statistics for high impact rare variants on Ashkenazi Jewish IBD cohort (Variants with $P < 0.05$ are displayed). Logistic regression model-based association analyses on all AJ IBD cases and controls. CHR, chromosome number. SNP, Single nucleotide polymorphism. BP, base position (GRCh 37). ID, RS ID for SNPs. Alt allele, alternative allele. OR, odds ratio. MAF, minor allele frequency. All P values are unadjusted, the statistical test is two-sided.

File Name: Supplementary Data 5
Description: List of known IBD-related genes used for gene prioritizations and pathway analyses.

File Name: Supplementary Data 6
Description: Summary of top candidate IBD genes from each pathway and functional enrichment analyses. HGC, Human Gene Connectome (http://lab.rockefeller.edu/casanova/HGC). ToppGene, (https://toppgene.cchmc.org/). IPA, Ingenuity Pathway Analysis. GIANT-Global, Genome-wide Analysis of gene Networks in Tissues, global tissue (http://giant.princeton.edu/)

File Name: Supplementary Data 7
Description: Gene prioritization by counting shared IBD genes in pathways and gene modules

Innate-GO, InateDB Gene Ontology analysis (https://www.innatedb.com/). Innate-Pathway, InnateDB pathway analysis. IPA, Ingenuity Pathway Analysis. NETanalyst, (https://www.networkanalyst.ca/). HGC, Human Gene Connectome.

File Name: Supplementary Data 8
Description: LD information between ICAM1 top variant and 3 IBD-associated variants in TYK2 result from Joint analysis. A joint analysis has been done on ICAM1 lead SKAT SNP rs142682313 and three IBD-associated variants among TYK2 (rs34536443, rs35018800, and rs12720356) using our AJ WES cohort. As shown in Supplementary Table 8, no LD pairs were identified across the four variants. R2 is used to represent the magnitude of LD.

File Name: Supplementary Data 9
Description: Joint association analysis and conditional analysis for ICAM1 top variant on three TYK2 variants. The independent effects of ICAM1 lead SNP (rs142682313) have been conditioned by three TYK2 variants (rs34536443, rs35018800, and rs12720356) using GCTA-COJO, the results indicating that the ICAM1 IBD variant is independent of the TYK2 variants. Conditionally dependent pairs of variants were defined as those whose conditioned P-values were at least an order of magnitude less significant than the individual single-point P-values. All P values are unadjusted, the statistical test is two-sided.

File Name: Supplementary Data 10
Description: Linkage disequilibrium analysis on INPP5D lead SNP rs574989226. A linkage disequilibrium analysis has been performed on INPP5D lead SNP rs574989266 using all AJ samples. The potential LD SNPs have been sought within 1Mb of rs574989266, a distance that fully covers the region of the ATG16L1 gene. R2 is used to represent the magnitude of LD.

File Name: Supplementary Data 11
Description: Association statistics for 46 high impact rare variants in 11 significant IBD genes identified by all pathway analyses. Association statistics for 68 high impact rare variants in 11 IBD-associated genes. Descriptions for the column IDs :CHR, chromosome number. SNP, Single nucleotide polymorphism. BP, base position (GRCh 37). ID, RS ID for SNPs. Alt allele, alternative allele. OR, odds ratio. MAF, minor allele frequency. Test ADD, genotypic model testing additivity. NMISS, non-missing individuals being used in association test. Hardy_P, the P values derived from Hardy-Weinberg equilibrium test. Missing (case, control), missing rate of variants in IBD cases and controls. VQSR, the indicator after running Variant Quality Score Recalibration. VQSLOD, the confidence score for running VQSR. The statistical test is two-sided.

File Name: Supplementary Data 12
Description: Average log expression of each gene across 31 cell types and differential expressions in CD and UC using bulk RNA-seq analyses. A. Average log expression of IBD genes across 31 cell types. Row names are 11 IBD genes identified from pathway analyses. Column names are cell types annotated from cell clusters. B. Differential expressions in CD and UC using bulk RNA-seq analyses. Row names are significant genes identified from gene-level association analysis. Columns are log2 Foldchange and P value in differential expression

analyses for CD and UC respectively, 125 of the 127 significant genes having available expression values are displayed.

File Name: Supplementary Data 13
Description: Raremetalworker results at variant level for dataset 1. Raremetalworker results at variant level for dataset 1. All P values are unadjusted, the statistical test is two-sided.

File Name: Supplementary Data 14
Description: Raremetalworker results at variant level for dataset 2. Raremetalworker results at variant level for dataset 2. All P values are unadjusted, the statistical test is two-sided.

File Name: Supplementary Data 15
Description: Meta analyses using high impact rare variants on IBD. Group name, gene symbols. NUM_VAR, number of variants being tested. VARs, IDs of variants have been aggregated into each gene. Min-MAX_AF, the minimal and maximum allele frequencies in cohorts.

File Name: Supplementary Data 16
Description: Gene level Phenome-wide association studies on 11 top genes using Mount Sinai BioMe Biobank whole exome sequencing samples. Code interpretation, clinical modification of ICD-10 WHO International Classification of Diseases. P.value, P value from SKATO analyses using high impact variants from IBDGC AJ cohort. N.Marker.Test, non-missing and non-monomorphic sites among the cases and controls for the genes. Carriers, number of samples carrying mutations in cases and controls. The statistical test is two-sided.

File Name: Supplementary Data 17
Description: QC metrics for genetically identified AJs in this study.

File Name: Supplementary Data 18
Description: Conditional analysis for gene-level PheWAS results on the nearby significant common variants around the rare variants. Conditional analysis for gene-level PheWAS results on the nearby significant common variants around the rare variants. The statistical test is two-sided.
