# Supplementary material for: Identifying high-impact variants and genes in exomes of Ashkenazi Jewish inflammatory bowel disease patients
Source: Nat Commun. 2023 Apr 20;14:2256. doi: 10.1038/s41467-023-37849-3 (PMC10119186; doi:10.1038/s41467-023-37849-3)
Supplement: Supplementary file 5 — Reporting Summary [file 41467_2023_37849_MOESM5_ESM.pdf]

nature portfolio

Corresponding author(s): Yuval Itan

Last updated by author(s): Mar 9, 2023

Reporting Summary

Nature Portfolio wishes to improve the reproducibility of the work that we publish. This form provides structure for consistency and transparency in reporting. For further information on Nature Portfolio policies, see our Editorial Policies and the Editorial Policy Checklist.

Statistics

For all statistical analyses, confirm that the following items are present in the figure legend, table legend, main text, or Methods section.

n/a	Confirmed	
☐	☒	The exact sample size (*n*) for each experimental group/condition, given as a discrete number and unit of measurement
☐	☒	A statement on whether measurements were taken from distinct samples or whether the same sample was measured repeatedly
☐	☒	The statistical test(s) used AND whether they are one- or two-sided<br>*Only common tests should be described solely by name; describe more complex techniques in the Methods section.*
☐	☒	A description of all covariates tested
☐	☒	A description of any assumptions or corrections, such as tests of normality and adjustment for multiple comparisons
☐	☒	A full description of the statistical parameters including central tendency (e.g. means) or other basic estimates (e.g. regression coefficient) AND variation (e.g. standard deviation) or associated estimates of uncertainty (e.g. confidence intervals)
☐	☒	For null hypothesis testing, the test statistic (e.g. *F*, *t*, *r*) with confidence intervals, effect sizes, degrees of freedom and *P* value noted<br>*Give P values as exact values whenever suitable.*
☒	☐	For Bayesian analysis, information on the choice of priors and Markov chain Monte Carlo settings
☐	☒	For hierarchical and complex designs, identification of the appropriate level for tests and full reporting of outcomes
☒	☐	Estimates of effect sizes (e.g. Cohen's *d*, Pearson's *r*), indicating how they were calculated

Our web collection on statistics for biologists contains articles on many of the points above.

Software and code

Policy information about availability of computer code

Data collection	No software were used to collect data.
Data analysis	Reads mapping and genotype calling: GATK, BWA.<br>Population structure analysis and relateness identification: KING(v2.1.6), STRUCTURE, PLINK(1.90).<br>Variant annotation and high impact variants filtration: VEP(v90), SnpEff(v4.2), Mutation Significance Cutoff, Human Gene Connectome.<br>Gene level association analysis: SKAT(v2.2.4); Variant level association analysis: PLINK(1.90).<br>Conditional analysis and joint association analysis: GCTA(v1.93).<br>Bulk RNA-seq analysis: TopHat2, DESeq2.<br>Single cell RNA-seq analysis: Cell Ranger (v2.1.0), Seurat (v3.0.1).<br>Phenome-wide association analysis: PheWAS, SKAT(v2.2.4).<br>Rare variant meta analysis: Raremetal, Raremetalworker.<br>Pathway analysis: IPA.

For manuscripts utilizing custom algorithms or software that are central to the research but not yet described in published literature, software must be made available to editors and reviewers. We strongly encourage code deposition in a community repository (e.g. GitHub). See the Nature Portfolio guidelines for submitting code & software for further information.

Data

Policy information about availability of data

All manuscripts must include a data availability statement. This statement should provide the following information, where applicable:

- Accession codes, unique identifiers, or web links for publicly available datasets
- A description of any restrictions on data availability
- For clinical datasets or third party data, please ensure that the statement adheres to our policy

The Single cell RNA-seq raw data used in this study are available at NCBI GEO database under accession code GSE134809 (https://www.ncbi.nlm.nih.gov/geo/query/acc.cgi?acc=GSE134809). The bulk RNA-seq raw data used in this study are available at NCBI GEO database under accession code GSE57945 (https://www.ncbi.nlm.nih.gov/geo/query/acc.cgi?acc=GSE57945). The Human Gene Connectome database for prioritizing the candidate genes is available at web-server (https://lab.rockefeller.edu/casanova/HGC). The Mutation Significance Cutoff database for selecting high impact variants is available at web-server (https://lab.rockefeller.edu/casanova/MSC). The gene-level and variant-level association data generated in this study are provided in the supplementary tables. The raw sequencing data and raw bulk RNA-seq data are protected and are not available due to data privacy laws, which can be available based on reasonable request to IBDGC consortium.

Human research participants

Policy information about studies involving human research participants and Sex and Gender in Research.

Reporting on sex and gender	NA
Population characteristics	Population characteristics were described in the PCA methods section.
Recruitment	NA
Ethics oversight	NA

Note that full information on the approval of the study protocol must also be provided in the manuscript.

Field-specific reporting

Please select the one below that is the best fit for your research. If you are not sure, read the appropriate sections before making your selection.

☒ Life sciences ☐ Behavioural & social sciences ☐ Ecological, evolutionary & environmental sciences

For a reference copy of the document with all sections, see nature.com/documents/nr-reporting-summary-flat.pdf

Life sciences study design

All studies must disclose on these points even when the disclosure is negative.

Sample size	Sample size was determined by the total number of exomes available in the study's cohort.
Data exclusions	Data exclusions were performed by analyzing QC metrics and PCA outliers. The aim is to filter out QC-failed samples and identify Ashkenazi Jewish samples to conduct the population specific analysis.
Replication	There is no wet lab experiment in this study. The significant IBD associated genes have been replicated in phenome wide association study using BioMe Biobank.
Randomization	This is a case-control study without random allocation of samples. The gender and PCs have been used as covariates to control gender bias and potential population stratification in every association tests.
Blinding	Not applicable.

Reporting for specific materials, systems and methods

We require information from authors about some types of materials, experimental systems and methods used in many studies. Here, indicate whether each material, system or method listed is relevant to your study. If you are not sure if a list item applies to your research, read the appropriate section before selecting a response.

Materials & experimental systems

n/a	Involved in the study
☒	☐ Antibodies
☒	☐ Eukaryotic cell lines
☒	☐ Palaeontology and archaeology
☒	☐ Animals and other organisms
☒	☐ Clinical data
☒	☐ Dual use research of concern

Methods

n/a	Involved in the study
☒	☐ ChIP-seq
☒	☐ Flow cytometry
☒	☐ MRI-based neuroimaging
