# Supplementary material for: Identifying high-impact variants and genes in exomes of Ashkenazi Jewish inflammatory bowel disease patients
Source: Nat Commun. 2023 Apr 20;14:2256. doi: 10.1038/s41467-023-37849-3 (PMC10119186; doi:10.1038/s41467-023-37849-3)
Supplement: Supplementary file 2 — Peer Review File [file 41467_2023_37849_MOESM2_ESM.pdf]

nature portfolio

Peer Review File

Identifying novel high-impact variants and genes in exomes of Ashkenazi Jewish inflammatory bowel disease patients

Open Access This file is licensed under a Creative Commons Attribution 4.0 International License, which permits use, sharing, adaptation, distribution and reproduction in any medium or format, as long as you give appropriate credit to the original author(s) and the source, provide a link to the Creative Commons license, and indicate if changes were made. In the cases where the authors are anonymous, such as is the case for the reports of anonymous peer reviewers, author attribution should be to 'Anonymous Referee' followed by a clear attribution to the source work. The images or other third party material in this file are included in the article's Creative Commons license, unless indicated otherwise in a credit line to the material. If material is not included in the article's Creative Commons license and your intended use is not permitted by statutory regulation or exceeds the permitted use, you will need to obtain permission directly from the copyright holder. To view a copy of this license, visit http://creativecommons.org/licenses/by/4.0/.

REVIEWER COMMENTS
Reviewer #1 (Remarks to the Author):

The authors present an exome-wide association study of inflammatory bowel disease in the Ashkenazi Jewish (AJ) subset of a large case-control cohort. The sample is relatively small (just under 2000 total cases) compared to other sequencing studies in general IBD, but is relatively large for a minority ethnicity study. The specific population is of great interest to the field given the higher burden of disease in the Jewish population, so this study is a welcome addition.

The authors combine gene-burden association data with a series of bioinformatic analyses to suggest a set of genes where they believe rare coding variation plays a role in the onset of disease in the Ashkenazi Jewish population. The bioinformatic prioritisation is based, in large part, on similarity (functional, network, etc) to known common IBD risk genes. The approach seems, at least in theory, a relatively robust way of identifying potentially associated genes, though it also seems to bias the results somewhat to known genetic risk pathways and limit the chances to discuss dramatically new or surprising things. This design is also, as far as I can see, not well suited to answering the question of whether these associations are specific (or have a larger effect size) in the AJ population compared to non-AJ Europeans. The authors attempt validations of these genes based on gene expression data, showing that these associations tend to be differentially expressed in IBD patients compared to controls (though, given the bioinformatic prioritisation approach, this is probably not surprising), and test their associations with other traits in the general population. The authors also demonstrate that machine-learning-based genetic risk prediction can be used to distinguish cases and controls using this data, though I think this section could be made more useful by making formal statistical comparisons (either between different methods/scores, between AJ and non-AJ individuals, etc). Overall, while the authors have some limitations due to low power and the analysis approach used, the results are a useful addition to the field.

My biggest hesitation about this paper is that it seems as if there may be significant confounding in the data, for reasons I lay out below. This is compounded by the lack of a replication dataset, which means that the associations that are reported (some of which are very highly significant for such a small sample set) are difficult to trust. I would like to see more analyses done to reassure the reader of the robustness of the results.

I also have a number of other specific issues with the analyses and claims made in the paper, that I outline below. I believe that most of these should relatively easily addressable, though some (e.g. the addition of non-AJ comparator associations) may be more difficult, depending on what data the authors have to hand.

Major issue on confounding:

As mentioned above, my biggest reservation about this paper is the possibility that confounding, introduced by technical sequencing issues or population structure, could be generating false positives in the association analysis. The absence of a replication set makes it very important that the reader can trust the primary analysis results, and I did not feel, in their current state, that the results inspired that level of trust.

1. There are large numbers of associations, including at the single variant level, that are highly significant. This looks like at least 15 in the all-IBD single-variant analysis, by my count, based on the Manhattan plot in Figure S5. This seems like a suspiciously large number for a small case-control study (this is approximately the same number of genome-wide significant hits as were found in the Sazonovs et al exome preprint, which had >15x the sample size, in the general IBD population). I suspect that the authors do not fully trust these association results themselves, as there are very strong associations (e.g. in SPAG11B) that are not even mentioned in the main text. The authors already note another association NCF1, which seems to show potentially artifactual differences in allele

frequency based on other datasets.
2. The primary association tests (at a gene or variant level), as far as I can tell, do not control for population stratification or for technical differences between the two data releases, both of which appear to be significantly confounded with case-control status. When the authors DO control for differences in between the two data releases, by splitting out the two datasets and meta-analysing them, the results change dramatically. For instance, three of the most significant genes in the (non-batch controlled) primary CD analysis, OR51A4, SPAG11B and NCF1, are completely flat in the (batch-controlled) CD meta-analysis. This is also true of essentially all of the UC associations. This seems to me to be clear evidence either of strong confounding, or of extreme sensitivity of the results to the other slight differences in the analysis method.
3. I could not see any clear details on how the technical quality of the sequencing, and in particular, potential biases between cases and controls, were assessed. This is particularly important as diagnoses are not balanced between the two datasets, and thus any technical differences between the two datasets will risk introducing false positives. Were QC statistics (coverage, % mapping, % on target, etc) comparable between cases and controls, and between the two batches?
4. Based on the PCAs (Figures 1B, S1 and S2), there appears to be significant heterogeneity within the "AJ" group. It isn't clear exactly how heterogeneous the finally selected sample set was, or whether the PCs or ancestry proportions differed between the two datasets or between cases and controls, but this needs to be investigated and controlled for in the association analyses.
5. The authors do not report any negative control analyses that would reassure the reader that false positives are under control (though they may have carried these out, I know that authors do not always report them). In particular, I would like to see A) a control-vs-control association analysis across the two separate datasets (i.e. testing for differences in the controls from dataset 1 and dataset 2), and B) a gene-level analysis of synonymous variants (i.e. replicating the high-impact analysis, but for low-impact variants), to demonstrate that neither of these produce false positives.

Other substantial comments:

- This section: "To this end, we performed Genome-wide Complex Trait joint and conditional analyses (GCTA-COJO) with ICAM1 lead SNP and three IBD-associated sites in TYK2, both of which suggested it to have independent protective effects against IBD (Supplementary Table 8 and 9)." looks the wrong way around to me, this shows that the TYK2 variant rs12720356 is independent of the ICAM1 variant, whereas the text says that the ICAM1 variant is independent of TYKY2. The authors should test this the other way around (i.e. test the ICAM1 variant conditional on the TYK2 variants).

- The authors state: "Five variants in different genes passed a Bonferroni-corrected P-value of 9.09×10-4 (=0.05/55)". This is entirely inappropriate, these p-values have been (indirectly, via the gene-level test) pre-selected for signifince and thus correcting for the 55 variants (rather than the 100,000 variants initially screened) will no longer guarantee family-wise error rates.

- The authors should give the version of RAREMETAL that was used. If the version was 4.14.0 or 4.14.1, a bug was discovered in these versions that gives false positives for certain tests, which should be checked.

- The text implies that these rare variants are more common in the AJ population ("disease related rare variants are highly enriched"), but I could not find any explicit testing of this for the genes under study here (we know that it is true in certain cases, such as NOD2, but we don't know if it is true for the novel genes that the authors propose).

- There is a more general issue here about not having comparisons to non-AJ associations, which limits the extent to which these results can be interpreted as AJ results (as opposed to just reflecting general IBD results). Is there a reason that the authors do not provide association statistics in the non-AJ (or general IBD) population for these loci, and test for heterogeneity between AJ and non-AJ effect sizes?

- The PheWAS does not seem like it was done conditional on the already-known common variant associations around LRRK2, INPP5D, ICAM1, so these cannot be properly seen as replications of the new rare variant associations (as opposed to just bleed-through from the common variant associations).

- The PRS predictions, while a nice addition, are missing vital information to allow us to interpret the results. Firstly, the authors need to add confidence intervals to the AUC and do some reclassification accuracy tests, as it is possible that all of these predictive methods are essentially equivalent and the differences are just due to sampling noise. Secondly, if the authors wish to make conclusions specific to the AJ population, it would be good to use the PRS from general (AJ + non-AJ) IBD from the latest meta-analyses (de Lange et al, I think), to see if having AJ-specific data increases accuracy compared to using general IBD data, and to run the analysis on some non-AJ samples, to test whether predictive accuracy differs depending on ancestry.

Minor comments:

- The statement on p2 that genes were "validated" in RNA-seq data seems too strong. The associations were not validated, they were just given further biological plausibility.

- A TLR4 associations with CD have been described before. Is the TLR4 association in this paper independent of the previously described TLR4 coding variant in CD (rs4986790, described in PMID: 26974007)?

- There seems to be some contradiction in the section on rs574989226/INPP5D. The text states "the most significant variant in INPP5D: rs574989226 ($P = 0.011$)", but then in the conditional analysis section it states "The significance of rs574989226 only slightly changed after the GCTA-COJO conditional test using our AJ cohort (conditioned, $P = 6.9 \times 10{-}3$; unconditioned, $P = 8.8 \times 10{-}3$ from GCTA-COJO)". Is the unconditional p-value 0.011 or 0.0088?

- The authors should provide site and genotype quality scores (including the VQSLOD and VQSR input fields, as well as the missingness, differential missingness, hardy-weinberg p-value, etc) for the variants in Table S11.

- The output given in Supp Tables 13 + 14 are difficult to understand, please fully describe all columns in the table legend. Please also give the input summary statistics for each of the individual datasets.

- The legend of Figure S2 incorrectly refers to Figure 2a (presumably this should be Figure 1b).

Reviewer #2 (Remarks to the Author):

Using an exome sequencing approach the authors have identified 7 novel IBD-causing genes in 4,974 genetically identified AJ subjects. This is an organized and thoughtful association analysis pipeline followed up with RNASequencing to validate the identified genes from the association analyses. My specific comments include:

1) There are many supplementary tables that are not referenced in the results or methods. It is confusing to parse through all these when not cited in the primary body. As an illustration of the confusion - Supplementary Table 2 is the first table one would expect as that is the primary SKAT-O result, this should then connect to the table with the single-SNP results and then followed by the 9 genes identified from the pathways? Please remove tables not referenced or reference them in the submission.

2) Why are single variant tests done in both SKAT-O as well as logistic regression models (reflected in Sup Table 5)? Also I am missing the point of the single variant tests where is is framed as 'To examine the contribution of variants within the significant genes' but then the single variants are evaluated at a GWAS threshold? If this is really to assess the contribution of the single variants to the genes identified through the gene-based approach, then the individual variants should not be penalized for GWAS thresholds.

3) Why only the 9 genes identified with the pathway approach further prioritized. The rationale to the pathway approach was "Since biologically relevant genes may not display genome-wide significance at the gene level due to genetic heterogeneity, we additionally applied pathway enrichment and biological relatedness approaches to identify biologically plausible IBD-causing genes from the SKAT-O significant genes." One would argue that under this rationale the final set of genes for prioritization should in fact be the union of those identified at exome-wide thresholds (n=15 genes) AND the 9 from pathways, and not limited to only those 9 from the pathway.

4) Why was GCTA-COJO used for conditional analysis when line level data is available? Would it not be more appropriate to model the SNPs jointly in the specific dataset that rely on summary statistics.

5) In the definition of the 'high impact' variant in "These 9 plausible IBD candidate genes harbor 55 high impact variants (Supplementary Table 1 and 11), it would seem that there are genes with only a single variant in the gene-based skat-o analysis. Please add #variants to Supp Table 1. Also why would just the 'top 5 ranking' SNPs be collapsed into a single set? The rationale seems unclear. Would be it more appropriate to consider the cumulative burden across all 9 genes as a single unit without filtering?

6) Please clarify the rationale to picking 268 differentially expressed genes because that number aligns with 268 skat-o identified genes with $p<0.01$. This seems arbitrary. RNASeq generally has the ability to identify more differential signal that association tests, and as such should not be held to a 'count' of top genes to align with the number passing the skat-o significance levels.

7) Please address significance thresholds. Early in results the exome-wide Bonferroni threshold is used to define 15 genes, this then switched to those with $p<0.01$ for the pathway approach to identify 9. However in the conclusion genes with $p<0.01$ are defined as 'significant'.

Responses to reviewer 1

The authors present an exome-wide association study of inflammatory bowel disease in the Ashkenazi Jewish (AJ) subset of a large case-control cohort. The sample is relatively small (just under 2000 total cases) compared to other sequencing studies in general IBD, but is relatively large for a minority ethnicity study. The specific population is of great interest to the field given the higher burden of disease in the Jewish population, so this study is a welcome addition.

The authors combine gene-burden association data with a series of bioinformatic analyses to suggest a set of genes where they believe rare coding variation plays a role in the onset of disease in the Ashkenazi Jewish population. The bioinformatic prioritisation is based, in large part, on similarity (functional, network, etc) to known common IBD risk genes. The approach seems, at least in theory, a relatively robust way of identifying potentially associated genes, though it also seems to bias the results somewhat to known genetic risk pathways and limit the chances to discuss dramatically new or surprising things. This design is also, as far as I can see, not well suited to answering the question of whether these associations are specific (or have a larger effect size) in the AJ population compared to non-AJ Europeans. The authors attempt validations of these genes based on gene expression data, showing that these associations tend to be differentially expressed in IBD patients compared to controls (though, given the bioinformatic prioritisation approach, this is probably not surprising), and test their associations with other traits in the general population. The authors also demonstrate that machine-learning-based genetic risk prediction can be used to distinguish cases and controls using this data, though I think this section could be made more useful by making formal statistical comparisons (either between different methods/scores, between AJ and non-AJ individuals, etc). Overall, while the authors have some limitations due to low power and the analysis approach used, the results are a useful addition to the field.

My biggest hesitation about this paper is that it seems as if there may be significant confounding in the data, for reasons I lay out below. This is compounded by the lack of a replication dataset, which means that the associations that are reported (some of which are very highly significant for such a small sample set) are difficult to trust. I would like to see more analyses done to reassure the reader of the robustness of the results.

I also have a number of other specific issues with the analyses and claims made in the paper, that I outline below. I believe that most of these should relatively easily addressable, though some (e.g. the addition of non-AJ comparator associations) may be more difficult, depending on what data the authors have to hand.

Major issue on confounding:

As mentioned above, my biggest reservation about this paper is the possibility that confounding, introduced by technical sequencing issues or population structure, could be generating false positives in the association analysis. The absence of a replication set makes it very important that the reader can trust the primary analysis results, and I did not feel, in their current state, that the results inspired that level of trust.

We greatly appreciate the Reviewer's thoughtful and thorough review, and their valuable comments and suggestions on our manuscript, which have helped us to improve it significantly. In response to the comments, we performed a major revision of our study and checked for potential confounding factors. Please see our responses to each specific comment below.

1. There are large numbers of associations, including at the single variant level, that are highly significant. This looks like at least 15 in the all-IBD single-variant analysis, by my count, based on the Manhattan plot in Figure S5. This seems like a suspiciously large number for a small case-control study (this is approximately the same number of genome-wide significant hits as were found in the Sazonovs et al exome preprint, which had >15x the sample size, in the general IBD population). I suspect that the authors do not fully trust these association results themselves, as there are very strong associations (e.g. in SPAG11B) that are not even mentioned in the main text. The authors already note another association NCF1, which seems to show potentially artifactual differences in allele frequency based on other datasets.

We thank the Reviewer for this important comment. The main focus of our current study was to improve our understanding of the missing heritability of IBD by identifying candidate genes in a high-risk group, the Ashkenazi Jewish population, rather than performing exome-wide associations in a larger multi-ethnic cohort. We employed high-impact rare variants to identify putative IBD-associated genes and prioritized 11 of them using pathway analyses and biological function proximity calculations. We agree with the Reviewer that there was the potential to identify false-positive associations in the study (as of course there is in any gene association study). Therefore, following the Reviewer's helpful recommendations, we updated our variant filtration criteria to further reduce the likelihood of false-positives. We also performed additional analyses so as

to provide sample- and variant- level quality control measures. Please see response #3 for a detailed explanation of our quality control process.

2. The primary association tests (at a gene or variant level), as far as I can tell, do not control for population stratification or for technical differences between the two data releases, both of which appear to be significantly confounded with case-control status. When the authors DO control for differences in between the two data releases, by splitting out the two datasets and meta-analysing them, the results change dramatically. For instance, three of the most significant genes in the (non-batch controlled) primary CD analysis, OR51A4, SPAG11B and NCF1, are completely flat in the (batch-controlled) CD meta-analysis. This is also true of essentially all of the UC associations. This seems to me to be clear evidence either of strong confounding, or of extreme sensitivity of the results to the other slight differences in the analysis method.

We thank the Reviewer for making this important point, which we followed strictly in the revision of our manuscript. We have addressed issues arising from the potential for confounding factors resulting from the population structure of AJs, heterogeneity between data releases and variant-level sequencing artifacts. To ensure that we obtained high quality samples and variants, we applied more stringent filters. In addition to eliminating possible confounding factors, we included the first 10 principal components as covariates in the variant- and gene- level association tests to overcome the impact of any remaining population stratification on the results. In the revised manuscript, we observed that inflation of the collapsing analyses has been successfully controlled, and more reliable results were obtained by following the suggestions of both reviewers. Some previous false positive results were likely derived from sequencing artifacts, and these have been ruled out in our updated results. We found that the results obtained were less likely to be confounded by population structure (please see response #4). After correcting for potential sequencing artifacts at the sample- and variant-level, we did not observe any obvious inflation in tests of controls *vs*. controls and of using synonymous variants only (please see response #5). As the updated high-quality samples and variants are fundamental to this study, we thoroughly revised every section where potential artifacts might have been implicated. In the updated results, 11 genes have been identified as IBD-associated genes by statistical testing as well as by 4 various pathway/biological function approaches. Please see the main text related to these genes:

'*We identified a final list of 11* genes *(EGR2, ICAM1, IL33, INPP5D, ITK, LRRK2, NOD2, PDGFD, RGS1, TLR4, and VDR) that occurred within the top-ranking results across all 4 pathway enrichment and biological relatedness approaches (IPA, ToppGene, GIANT and HGC). All of these genes have been reported as having pathogenic mutations in non-IBD diseases (ICAM1, ITK, LRRK2, NOD2 and INPP5D in primary immunodeficiency; EGR2 in systemic lupus erythematosus; PDGFD, IL33 and RGS1 in autism spectrum disorder; TLR4 in type 2 diabetes, gastritis and susceptibility to infectious diseases; VDR in Vitamin D-resistant rickets) in the Human Gene Mutation Database (HGMD) Professional version[12]. In summary, three genes, NOD2, LRRK2 and VDR are known IBD genes (Supplementary Table 7), whereas the other 8 are novel, and not yet formally implicated in IBD (Fig. 2, Supplementary Results).*

We then investigated the likely physiological relatedness of the 8 novel prioritized genes to IBD. Variants in ICAM1 and INPP5D are reported to be associated with primary immunodeficiencies in HGMD. ICAM1 is involved in mediating adhesive interaction between lymphocytes and endothelial cells, and has been recognized as a potential therapeutic target in IBD[13,14]. Since ICAM1 is located within 100kb of TYK2 (a gene known to be associated with IBD pathogenesis[15], we sought to determine whether the ICAM1 lead variant (rs142682313, OR=0.4, P = 7.16×10^{-04}) was conditionally independent of IBD-associated sites in TYK2. To this end, we performed Genome-wide Complex Trait joint and conditional analyses (GCTA-COJO)[16] with the ICAM1 lead SNP and three IBD-associated sites in TYK2, both of which suggested that the ICAM1 IBD variants act independently of the TYK2 variants (Supplementary Table 8 and 9). INPP5D encodes SHIP1 protein, whose expression level is significantly associated with IBD[17,18]. INPP5D resides in close proximity on chromosome 2q37.1 to another IBD gene, ATG16L1[19]. We therefore performed linkage disequilibrium (LD) analysis on the most significant variant in INPP5D, rs574989226, and demonstrated that there were no strong LD pairs identified between ATG16L1 and INPP5D (Supplementary Table 10). Additionally, we performed a conditional analysis on the well-described IBD variant rs2241880[20] in ATG16L1 to check the independence of rs574989226. The significance of rs574989226 only slightly changed after the GCTA-COJO conditional test using our AJ cohort (conditioned, $P_{con} = 1.29 \times 10^{-2}$; unconditioned, $P_{uncon} = 1.04 \times 10^{-2}$ from GCTA-COJO), which indicates that rs574989226 is independent from rs2241880.

Variants in EGR2 are associated with the autoimmune diseases, systemic lupus erythematosus and celiac disease. As a member of a zinc finger transcription factor family, EGR2 is known to display suppressive activity with regard to

CD4+ T cells, and control the production of inhibitory cytokines such as IL-10 and TGF-β1[21]. A previous study also revealed that the expression of EGR2 is upregulated in inflamed colonic biopsies when compared to healthy colon[22], suggesting that EGR2 is likely to be an IBD-associated gene. IL33 has long been considered to play an important role in intestinal immunity. IL33 and its membrane receptor ST2 act as critical regulators of inflammation[23,24]. TLR4 plays a key role as the hub of the immune response to microbes in the gut in IBD pathogenesis[25]. PDGFD, a differentially expressed gene in crypt-associated fibroblasts, has been reported to be significantly downregulated in the colonic mucosa of Crohn's disease patients[26]. Moreover, single cell analyses of Crohn's disease tissues revealed that γ δ T cells selectively expressed PDGFD[27], which indicates that PDGFD might play a role in IBD. Although there are no records of IBD for RGS1 in HGMD, RGS1 is a member of the regulators of G-protein signaling (RGS) family, which is considered to be a promising target for the treatment of gastrointestinal inflammation[28]. Gibbons et al. have shown that RGS1 expression is significantly higher in human gut T cells compared to T cells derived from peripheral blood and this difference can further increase in intestinal inflammation. More specifically, RGS1 mRNA is significantly elevated in T cells obtained from intestinal samples of CD and UC patients when compared with healthy controls. They have also demonstrated that RGS1 is a dominant regulator of T cell trafficking in the gut, and therefore it could be involved in the pathology of IBD[29].

IL-2-inducible tyrosine kinase (ITK) is primarily expressed in T cells, and is essential for proximal T cell receptor (TCR) signaling. Studies have shown that ITK is involved in the pathogenesis of autoimmune diseases, including rheumatoid arthritis, systemic lupus erythematosus, multiple sclerosis and IBD[30]. ITK harbors a variant (rs753847568, p.Val264Ile) associated with very early onset inflammatory bowel disease (VEO-IBD) according to the HGMD[31]. Since five of the identified IBD-associated genes have been implicated in primary immunodeficiency, which is closely linked with VEO-IBD, we used HGC to check the biological association of candidate genes with the list of known VEO-IBD-causing genes[32]. The analysis of known VEO-IBD-causing genes versus random gene sets yielded a P = 0.023 in 10,000 resampling iterations. Interestingly, these analyses indicated that the genetic basis of AJ IBD resembles that of IBD in young children under the model built using rare high impact mutations. Taken together, these findings demonstrated the strength of population-specific analyses in AJ. Therefore, all 8 novel genes described in this study are likely to have functional relevance to IBD.

Investigating the two well-known IBD genes prioritized in our analyses, NOD2 had higher significance in the CD-specific SKAT-O analysis ($P = 9.51\times10^{-14}$, Supplementary Fig. 3 and Supplementary Table 2) but was insignificant in the UC-specific analysis (P = 0.85) (Supplementary Fig. 4 and Supplementary Table 3) as expected, since NOD2[33] is not known to cause UC. The significance of LRRK2 demonstrated the same trend: LRRK2 was more significant in the CD-specific test ($P = 9.68\times10^{-4}$) and the IBD-specific test ($P = 2.53\times10^{-4}$), but showed no significance in the UC-specific test (P = 0.07). However, LRRK2 showed lower significance compared to NOD2, due to the number of nominal significant LRRK2 variants that were used in the SKAT-O test (LRRK2 has only one significant site among 14 high impact variants, whereas NOD2 has five significant sites among 14 high impact variants, see Supplementary Table 11). The NOD2 rs104895438 and LRRK2 rs34637584 variants have been shown to be enriched in the AJ population and their independence has been confirmed by a previous study[8] via conditional analyses, whereas the other variants have not previously been implicated in IBD.'

3. I could not see any clear details on how the technical quality of the sequencing, and in particular, potential biases between cases and controls, were assessed. This is particularly important as diagnoses are not balanced between the two datasets, and thus any technical differences between the two datasets will risk introducing false positives. Were QC statistics (coverage, % mapping, % on target, etc) comparable between cases and controls, and between the two batches?

We thank the Reviewer for this suggestion. Although the two WES datasets were processed in two different batches, both were sequenced at the same center, which should in principle have reduced the technical differences between them. In this revised version, we present all QC metrics that are available for us by means of raw data and VCF files. To evaluate potential differences between batches using the available data, we first calculated the average depth of each variant across the whole dataset. Then we compared the distributions of the results of the 13,845,445 overlapping variants. The results showed that the two datasets have similar sequencing depth distributions: the average depth of the overlapping variants in dataset 1 was 44.27, whereas it was 41.02 in dataset 2. Additionally, the Pearson correlation test revealed a strong correlation between the depths of the overlapping sites in the two datasets ($R = 0.95$, $P < 2.2\times10^{-16}$). We generated the following Figure 1 to present the results.

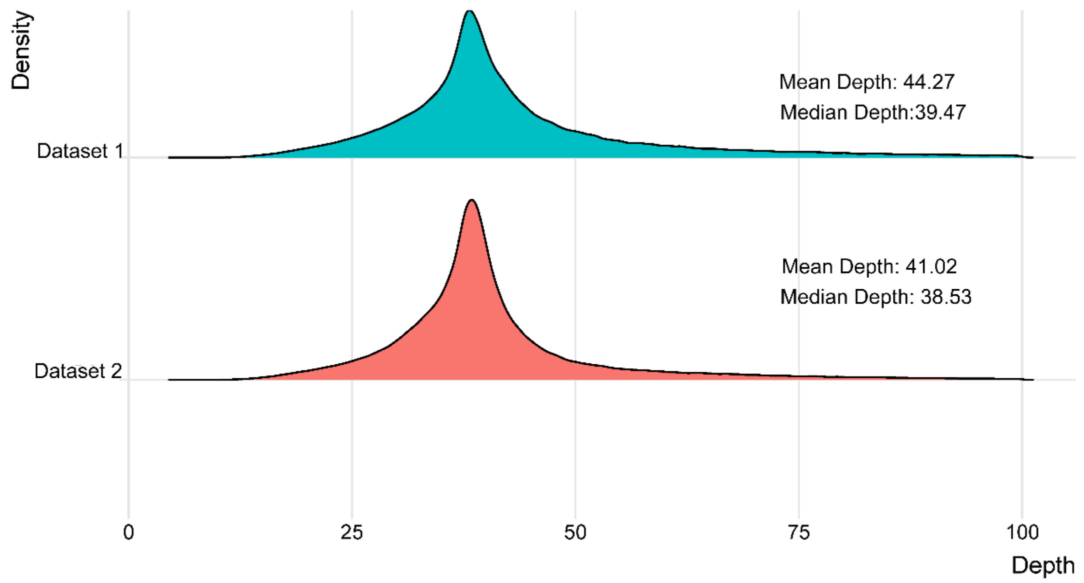

Figure 1. A density plot for the depth of overlapped variants between two datasets.

In addition to current QC filters, we applied additional filters so as to further eliminate the possibility of false-positive results. Specifically, at the variant level, variant sites were considered to be high-quality if they met the following criteria: (1) variants with a PASS filter status by Variant Quality Score Recalibration (VQSR). (2) variants with an average depth (DP) ≥10 and a genotype quality (GQ) ≥20 in all samples. (3) variants with alternate alleles that have DP ≥10 and a GQ ≥20 in at least one individual. (4) variants with a 'PASS' value in the FILTER column of the gnomAD v2.1 VCF file. In case-control analyses, we further excluded variants exceeding a missingness rate of 0.05 in cases, controls and the entire cohort. We also filtered out variants with high differential missing rate ($P < 1.0\times10^{-5}$). At the sample level, we obtained QC statistics for all samples by summarizing variants limited to the consensus coding sequence (CCDS) regions (Supplementary table 17). To minimize the potential bias that might arise from sequencing and variant discovery processes, we further excluded any samples falling outside of 4 median absolute deviations (MAD) from the median for any of the given metrics (PMID: 35255492): (1) the ratio of the number of heterozygous genotypes to the number of homozygous alternate genotypes; (2) the transition/transversion ratio of the passing bi-allelic SNP calls made at dbSNP sites (version 138, b37). (3) The insertion/deletion ratio of the indel calls made at dbSNP sites. The implementation of this procedure removed 101 samples from this study. We additionally re-ran admixture analysis on the two datasets and removed 427 AJ samples which have a relatively low AJ fraction compared to the lowest AJ fraction among the AJ reference panel (please see response #4 for details). In summary, the number of variants was reduced from 96,309 to 63,864 after applying these stringent filters to the SNP set. At the sample level, 521 additional samples were excluded from the initial 4,974 samples due to the aforementioned QC procedure; as a result, 4,453 QC-passed AJs comprising 1,494 samples from dataset 1 and 2,959 samples from dataset 2, and a total of 1,734 cases and 2,719 controls, remained in the analyses in our updated manuscript. We then checked the average depth of the samples for the overlapped variants in the two datasets and found that there was no significant difference between the two (44.3 and 44. 4 for dataset 1 and dataset 2, respectively, two-samples t test, $P = 0.72$). We were also able to evaluate the QC metrics of 3,387 samples (1,477 samples from dataset 1 and 1,910 samples from dataset 2), namely the percentage of aligned reads passing Illumina's filter (PF Reads Aligned %) and the mean target coverage, which were similar in dataset 1 and dataset 2, as well as in cases and controls (Table 1). We added sample-based metrics to the Supplementary Table 17. Therefore, we opted to perform the downstream analyses by selecting high-impact variants from the combined dataset.

Table 1. QC metrics obtained from raw data files

	**Dataset 1**	**Dataset 2**	**Cases**	**Controls**
#Samples	1,477	1,910	1,384	2,003
PF Reads Aligned %	99.3 ± 0.47	99.4 ± 0.46	99.4 ± 0.53	99.5 ± 0.41
The mean target coverage	85.1 ± 5.98	85.9 ± 4.58	86.4 ± 5.5	85.0 ± 5.0

4. Based on the PCAs (Figures 1B, S1 and S2), there appears to be significant heterogeneity within the "AJ" group. It isn't clear exactly how heterogeneous the finally selected sample set was, or whether the PCs or ancestry proportions differed

between the two datasets or between cases and controls, but this needs to be investigated and controlled for in the association analyses.

We thank the Reviewer for this recommendation. As we noted in response #2, we included the first 10 PCs as covariates (explaining 51.4% of the variance) in the analyses to control for population stratification. We also generated two additional PCA plots demonstrating the level of genetic homogeneity in cases and controls within both datasets. The distributions of samples in both plots did not reveal any population stratification in terms of the datasets (Figure 2A) or case-control status (Figure 2B) and were consistent with those of the AJ reference samples.

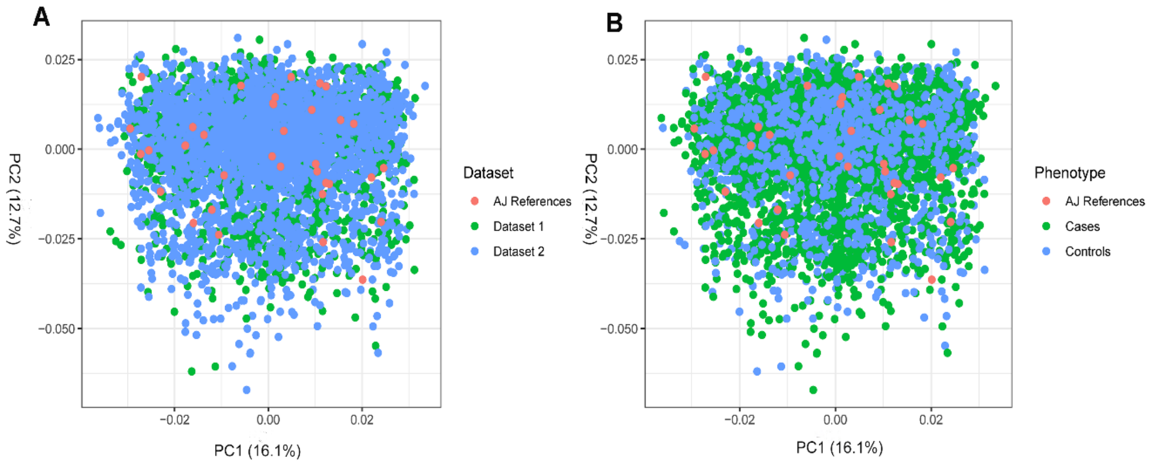

Figure 2. Principal component analysis of genetically identified Ashkenazi Jewish samples (compared to AJ references). Individuals are color coded based on either source of dataset (A) or case control status (B).

We then removed AJ reference panel samples and repeated the PCA on genetically identified AJs only. We still did not observe any obvious population stratifications biased by either dataset (Figure 3A) or phenotype (Figure 3B).

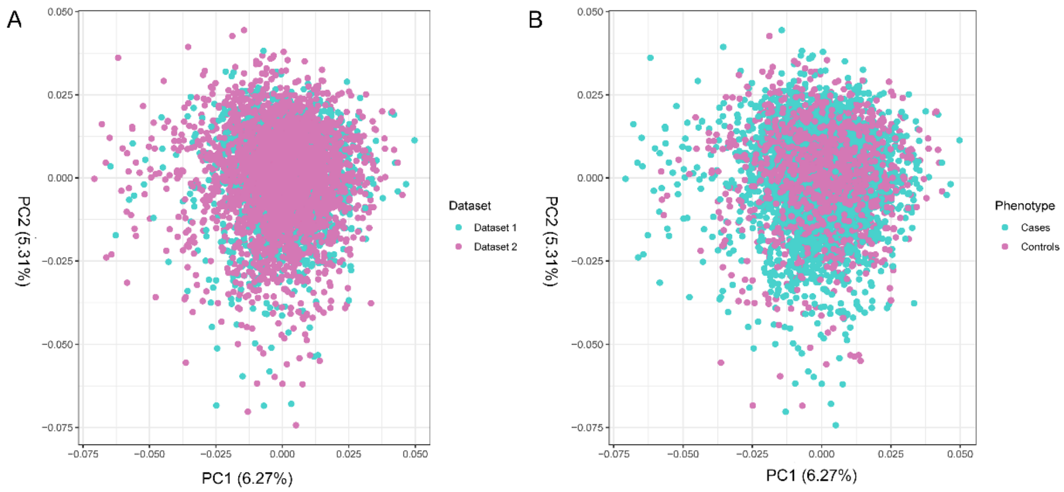

Figure 3. Principal component analysis of genetically identified Ashkenazi Jewish samples (without AJ references). Individuals are color coded based on the source of dataset (A) or case control status (B).

We additionally ran admixture analyses to evaluate ancestral contributions of genetically identified AJs and compared them with those from the Ashkenazi Jewish reference panel and the CEU population (Utah Residents with Northern and Western European Ancestry) from the 1,000 Genomes Project. Analyses with k from 2 to 6 were run for the combined dataset in which $k = 2$ resulted in the lowest cross-validation error (Figure 4).

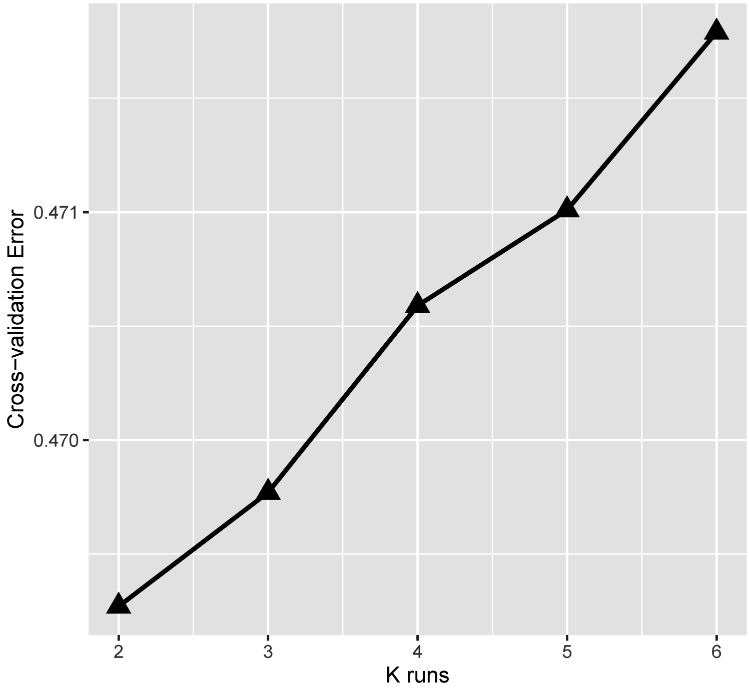

Figure 4. Cross-validation errors for 2nd round of AJ identification by comparing all AJ candidates to AJ reference panel and European panel, k = 2 gave the lowest cross-validation error.

The results indicated that the genetically identified AJs from the two datasets had ancestry fractions closely matched with the AJ reference panel. Overall, we did not observe any remarkable genetic heterogeneity in the two AJ datasets (Figure 5) or in the combined dataset. Nevertheless, we agree that the remaining population heterogeneity originating from varying AJ fractions within each dataset for our case-control analyses needs to be carefully controlled. In addition to the previous AJ identification procedure which had been performed within each dataset, we also integrated two datasets with the AJ reference panel and CEU reference panel, and then performed admixture analyses once again. We opted to use the lowest AJ fraction in the AJ reference panel as the threshold for the fraction of AJ ancestry, which was a trade-off between the power of the analyses and the homogeneity of AJ fractions. Finally, 427 AJ samples were removed from the current study. Moreover, we updated our case-control analyses using the first 10 principal components as covariates to strictly control the potential population stratification within the AJ group according to the Reviewer's suggestions.

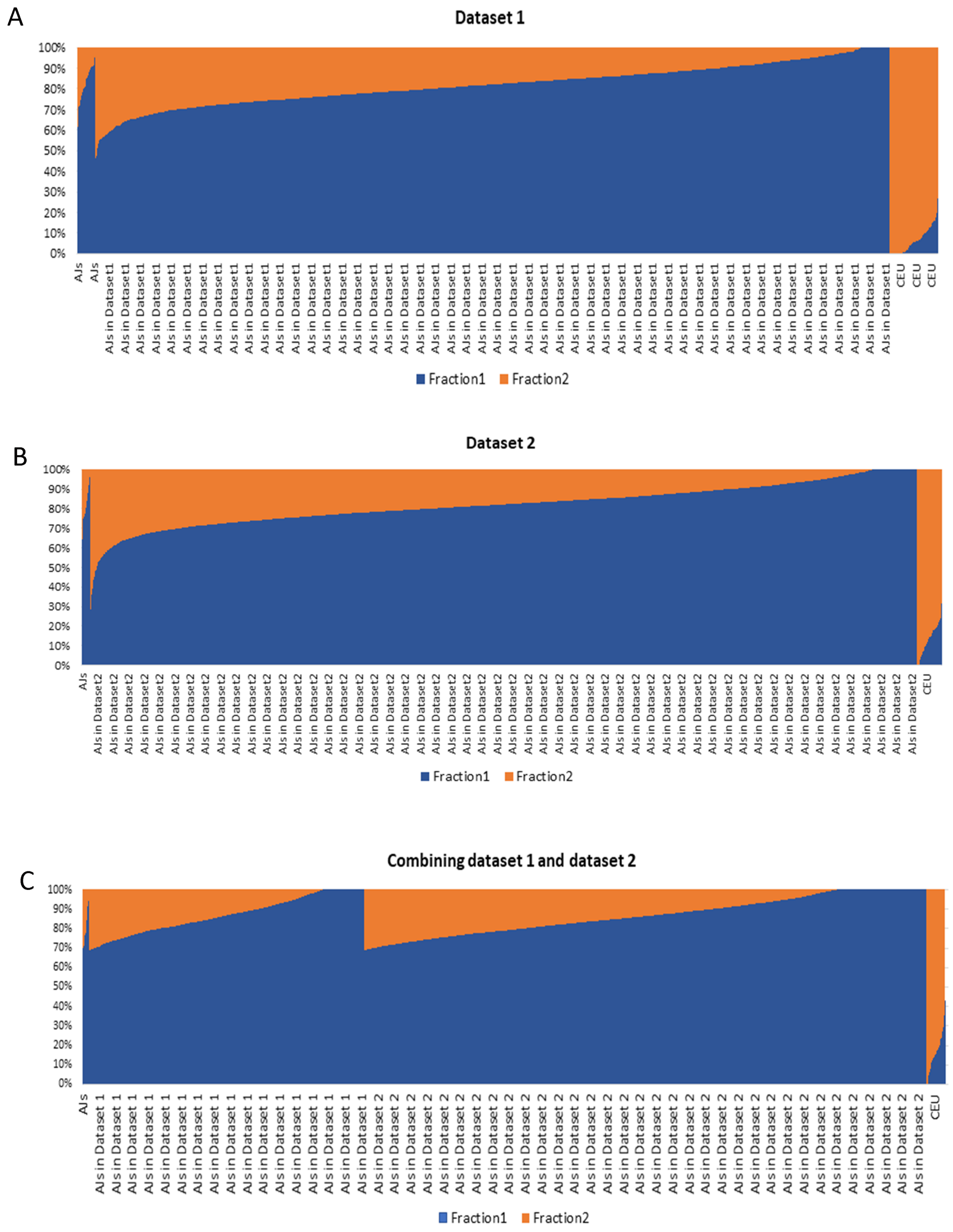

Figure 5. The admixture analyses for the A) Dataset 1, B) Dataset 2, and C) combined dataset together with the samples from the AJ reference panel and the CEU population from the 1000 Genomes Project after further filtering samples with relatively low AJ fraction (AJ fraction < 0.69, which is the lowest AJ fraction among the AJ reference panel).

5. The authors do not report any negative control analyses that would reassure the reader that false positives are under control (though they may have carried these out, I know that authors do not always report them). In particular, I would like to see A) a control-vs-control association analysis across the two separate datasets (i.e. testing for differences in the controls from dataset 1 and dataset 2), and B) a gene-level analysis of synonymous variants (i.e. replicating the high-impact analysis, but for low-impact variants), to demonstrate that neither of these produce false positives.

We are grateful for these helpful suggestions. To address them, we first performed control-*vs*.-control association analysis using the high-quality, high impact variants. The SKAT-O analysis was conducted using 436 controls from Dataset 1 *vs*. 2,283 controls from Dataset 2. The results are depicted below:

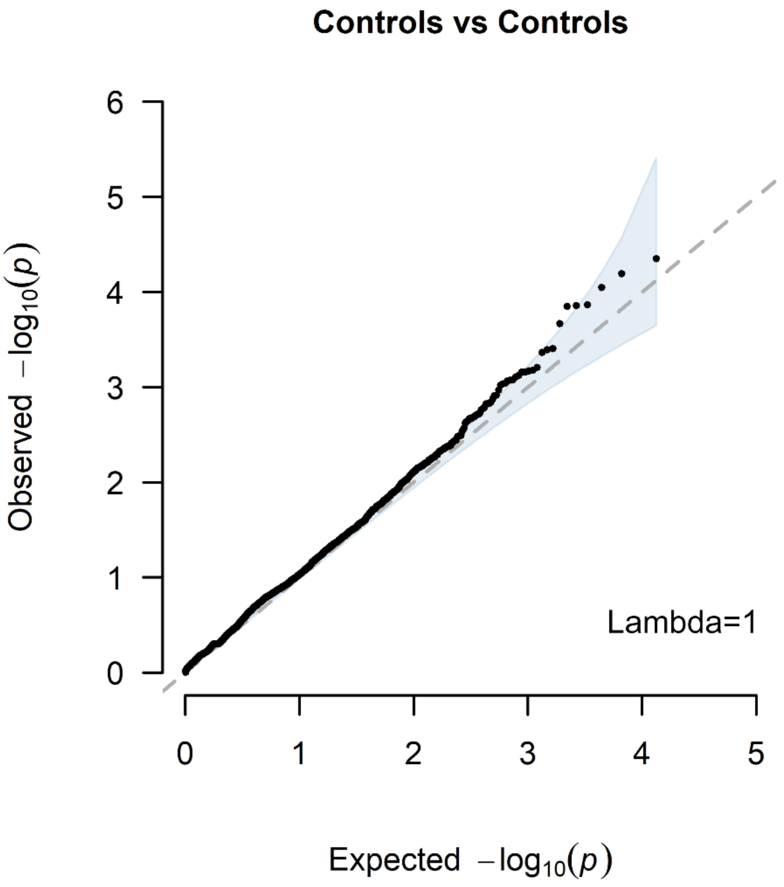

Figure 6. Q-Q plot for SKAT-O analysis of controls vs controls.

As shown in the Q-Q plot (Figure 6), we did not observe any evidence for genomic inflation when comparing Dataset1 controls *vs.* Dataset 2 controls.

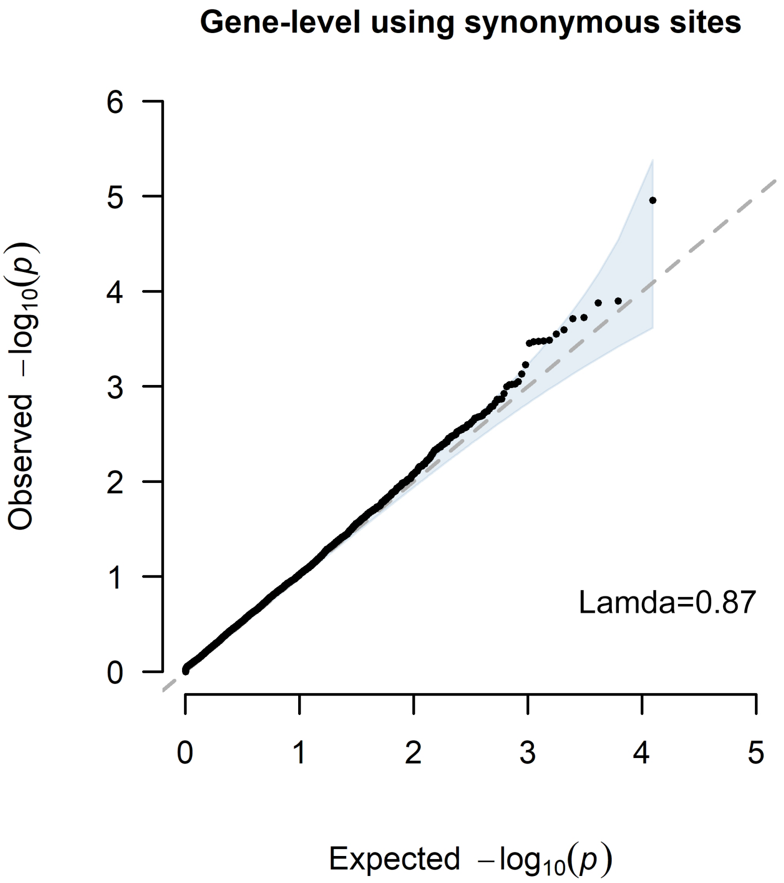

Figure 7. Q-Q plot for collapsing synonymous variants for IBD *vs.* controls.

We then ran SKAT-O for IBD cases *vs.* controls including only synonymous variants (Figure 7). Again, we did not detect any genomic inflation. We added the following text to the Methods section:

'*We performed a collapsing analysis that considered only synonymous variation as a neutral model to estimate the degree of inflation due to population substructure or possible technical artifact. We also performed a collapsing analysis of controls-vs.-controls using high-impact rare variants to check the possible heterogeneities in controls between two datasets. Neither analysis indicated a significant level of inflation in the results (Supplementary Fig. 12 and 13).*'

The Figures for the controls *vs.* controls and synonymous variant analyses have been added as Supplementary Figures 10 and 11, respectively.

Other substantial comments:

- This section: "To this end, we performed Genome-wide Complex Trait joint and conditional analyses (GCTA-COJO) with ICAM1 lead SNP and three IBD-associated sites in TYK2, both of which suggested it to have independent protective effects against IBD (Supplementary Table 8 and 9)." looks the wrong way around to me, this shows that the TYK2 variant rs12720356 is independent of the ICAM1 variant, whereas the text says that the ICAM1 variant is independent of TYKY2. The authors should test this the other way around (i.e. test the ICAM1 variant conditional on the TYK2 variants).

We thank the Reviewer for this helpful suggestion. We double checked our original test procedure. The process we followed was as the Reviewer mentioned. We have updated the conditional analyses to test whether the *ICAM1* site is independent from the *TYK2* variants. For clarification, we rephrased the sentence to read 'both of which suggested the *ICAM1* IBD variant is independent of the *TYK2* variants.'. Please see the results in updated Supplementary Table 9:

Conditional analysis for ICAM1 lead variant rs202183386		Beta (b) and P-value (p) before conditioning		Beta and P-value after conditioning	
Sites conditioning on	RSID for TYK2 sites	Beta	P-value	Beta-C	P-value-C
TYK2 site 1	rs34536443	-0.183976	0.000376703	-0.183777	0.000388425
TYK2 site 2	rs35018800	-0.183976	0.000376703	-0.183822	0.000387135
TYK2 site 3	rs12720356	-0.183976	0.000376703	-0.177359	0.000617222

- The authors state: "Five variants in different genes passed a Bonferroni-corrected P-value of 9.09×10-4 (=0.05/55)". This is entirely inappropriate, these p-values have been (indirectly, via the gene-level test) pre-selected for signifince and thus correcting for the 55 variants (rather than the 100,000 variants initially screened) will no longer guarantee family-wise error rates.

We agree with the Reviewer and have revised the Discussion based on the updated results as follows:

'*These 11 plausible IBD candidate genes harbor a total of 46 high impact variants (Supplementary Table 11). To test the burden of the significant SNPs ($P < 0.05$) located within the IBD candidate genes, we aggregated all significant SNPs from each IBD candidate gene into a single SNP set; the mutation carrier frequency in cases was 15.74% compared to 9.26% in controls, with an odds ratio (OR) of 1.83 ($P = 8.78\times10^{-11}$ by chi-squared test) despite two protective sites that are included in the analyses.*'

- The authors should give the version of RAREMETAL that was used. If the version was 4.14.0 or 4.14.1, a bug was discovered in these versions that gives false positives for certain tests, which should be checked.

We thank the Reviewer for pointing this out. The version of RAREMETAL used was 4.15.1, and this is now specified in the Methods. We also updated our RAREMETAL analysis. The meta-analysis results have been revised based on both our new sample- and variant-level QC filtration; please see the related content:

'*Of the 13,289 genes that were investigated, only two genes, ZSCAN5B and NOD2, passed the Bonferroni-corrected threshold of $P < 3.76\times10^{-6}$ in IBD case-control meta-analysis (Supplementary Table 15). However, the function of ZSCAN5B in IBD is yet to be investigated in future studies. Following NOD2, though not passing Bonferroni-adjusted significance, BIN3 and DAGLA have relatively strong association with IBD. BIN3 is a tumor suppressor gene which, interestingly, has been found to be upregulated in the healed mucosa of UC patients when compared with non-healed inflamed mucosa[36]. DAGLA was suggested as a potential druggable target based on its increased expression in ulcerative pancolitis compared to healthy human colonic tissue[37]. ICAM1, LRRK2, NOD2, PDGFD and RGS1 were again identified as IBD-associated genes at the same significance level ($P < 0.01$) in the meta-analysis.*'

- The text implies that these rare variants are more common in the AJ population ("disease related rare variants are highly enriched"), but I could not find any explicit testing of this for the genes under study here (we know that it is true in certain cases, such as NOD2, but we don't know if it is true for the novel genes that the authors propose).

We thank the Reviewer for this comment. A previous study suggested that 34% of protein-coding alleles were significantly enriched in the AJ population (Rivas MA et al. Insights into the genetic epidemiology of Crohn's and rare diseases in the Ashkenazi Jewish population. PLoS Genet. 2018). We agree that this conclusion was not replicated in our study as we did not perform any explicit testing here. Therefore, we removed this sentence from the main text.

- There is a more general issue here about not having comparisons to non-AJ associations, which limits the extent to which these results can be interpreted as AJ results (as opposed to just reflecting general IBD results). Is there a reason that the authors do not provide association statistics in the non-AJ (or general IBD) population for these loci, and test for heterogeneity between AJ and non-AJ effect sizes?

We greatly appreciate these comments. The main focus of the current study lies in the evaluation of the genetic underpinnings of IBD in the AJ population given the AJ population has a high IBD susceptibility and high frequencies of protein coding alleles (https://doi.org/10.1371/journal.pgen.1008190). We plan to compare the genetic associations of IBD in the AJ and non-AJ populations in a future study.

- The PheWAS does not seem like it was done conditional on the already-known common variant associations around LRRK2, INPP5D, ICAM1, so these cannot be properly seen as replications of the new rare variant associations (as opposed to just bleed-through from the common variant associations).

We thank the Reviewer for pointing this out. Instead of running a traditional PheWAS at the variant level using common variants, we implemented a gene-level PheWAS method by examining the associations by collapsing high-impact rare variants to better understand the associations between high impact rare variants and their respective phenotypes. The results have now been updated as below:

'*Gene-level PheWAS were performed for the 11 candidate genes. In total, 1,569 phenotypes with at least 100 cases exhibited a phenome-wide significance level of 3.18×10^{-5}. The association between Parkinson's disease and LRRK2 was just above the phenome-wide significance level (G20, P = 2.21×10^{-6}). Previous analyses have demonstrated that LRRK2 can play important roles in both PD and IBD[1]. Here, the same set of high impact rare variants has been used for the analysis of both phenotypes; therefore, the results obtained may have indicated that the comorbidity of PD and IBD is driven by LRRK2 rare variants. Thus, NOD2 was the most relevant gene to IBD, being significantly associated with Crohn's disease of the small intestine (K50.00, P = 8.23×10^{-5}) and Crohn's disease (K50.90, P = 3.85×10^{-4}). Other than NOD2, the ICAM1 gene was found to be associated with ulcerative (chronic) pancolitis without complication (K51.00, P = 1.59×10^{-2}) and ulcerative colitis (K51.919, P = 3.0×10^{-2}) in BioMe. In addition to IBD, ICAM1 is associated with type 1 diabetes mellitus without complications (E10.9, P = 1.64×10^{-3}). It was already known that type 1 diabetes patients have a higher risk of developing inflammatory bowel disease[2,3], and that the ICAM1 gene is potentially associated with the comorbidity of both diseases. The other candidate genes did not display significant associations with IBD in the BioMe Biobank PheWAS analyses. This is probably because of the small number of high impact rare variants being covered in the tested exomes combined and because of the limited IBD sample size (678 IBD samples, including CD and UC) in BioMe.*'

- The PRS predictions, while a nice addition, are missing vital information to allow us to interpret the results. Firstly, the authors need to add confidence intervals to the AUC and do some reclassification accuracy tests, as it is possible that all of these predictive methods are essentially equivalent and the differences are just due to sampling noise. Secondly, if the authors wish to make conclusions specific to the AJ population, it would be good to use the PRS from general (AJ + non-AJ) IBD from the latest meta-analyses (de Lange et al, I think), to see if having AJ-specific data increases accuracy compared to using general IBD data, and to run the analysis on some non-AJ samples, to test whether predictive accuracy differs depending on ancestry.

We thank the Reviewer for these helpful suggestions, which allowed us to significantly improve our PRS analyses. We re-calculated all PRS using revised samples and variants, and then added 95% confidence intervals to each ROC curve.
We first tried to derive PRS from de Lange's meta-analyses. However, we only found 334 associated variants from the GWAS Catalog for the study (Study ID: GCST004133), of which only 25 had available effect size information. Given that the limited number of variants may be insufficient to generate a PRS representing the genetic background of non-AJs, we instead prepared a GWAS summary using summary statistics from the meta-analysis performed in Liu et al. (PMID: 26192919), which included 34,666 IBD cases and 34,872 controls of European descent. The GWAS summary statistics for European IBD were obtained from the International Inflammatory Bowel Disease Genetics Consortium (IIBDGC, https://www.ibdgenetics.org/). To compare these with the PRS derived from GWAS summary based on AJs, we applied the GWAS summary data of Europeans with IBD to calculate the PRS for half of the AJ samples in our dataset. The results showed that the non-AJ GWAS statistics are not as strong as the AJ GWAS statistics in predicting risk of IBD in the AJ population, which is consistent with what we anticipated. The AUCs were 0.66 and 0.73 for non-AJ and AJ specific predictions, respectively (Please see the figure below).
Overall, the conclusions drawn from the updated datasets are similar to our previous results, which indicate that inclusion of high impact rare variants can enhance the PRS model in making predictions pertaining to IBD.

We think that the possible effect of sampling noise on the results should be minimal because our AUCs were summarized from overall results where the PRS scores of all individuals were predicted once during the cross-validation procedure. Meanwhile, the reclassification approach (such as Net Reclassification Index) may not be appropriate to measure the incremental change from common or rare variants as it is a measure for evaluating the improvement in prediction performance gained by adding a marker to a set of baseline predictors. Nevertheless, we are blending common and rare variants into 7 predictors of PRS rather than adding them to the model as individual predictors. Thus, every predictor is actually a mixture of the effects of variants. Still, we employed the integrated discrimination improvement (IDI) to evaluate risk predictions from model using common plus rare variants and model using common variants based on different GWAS summary statistic. The results are described as below:

'*Lastly, we evaluated the performance of rare and high impact variants in identifying individuals at risk for IBD using PRS with a Random Forest machine learning classification algorithm. We first used LD-pred to calculate the polygenic risk score for each individual, then employed risk scores as features to predict the IBD status of individuals with a Random Forest machine learning algorithm. We compared models based on the risk score results from six combinations of two GWAS summary statistics (a previous GWAS on AJ IBD samples from Hui et al.[38]; the other GWAS on half of our AJ IBD samples) and three sets of variants (high impact rare sites, common variants and both sets combined). As shown in Fig. 3e, the combinations of GWAS summary statistics and SNP sets displayed comparable predictive power, which result in areas under the curve (AUC) ranging from 0.71 to 0.75, while high impact rare variants displayed slightly better predictive power compared to common variants under both GWAS statistic sets. The AUC of rare variants were 0.75 and 0.72 for Hui et al. and our GWAS statistics, respectively, whereas the AUC of common variants were 0.72 and 0.71, respectively. The integrated discrimination improvement (IDI) calculated by PredictABEL[39] was used to evaluate risk predictions from the model with common variants and the model with both common and rare variants. Using both common and rare variants in the model improved reclassification with an IDI of 1.00% for Hui's GWAS summary statistic, but decreased the reclassification with an IDI of 2.69% for the GWAS summary statistics generated by half of our dataset, which indicate that the effects of rare variants may require accurate assessment in risk prediction.*'

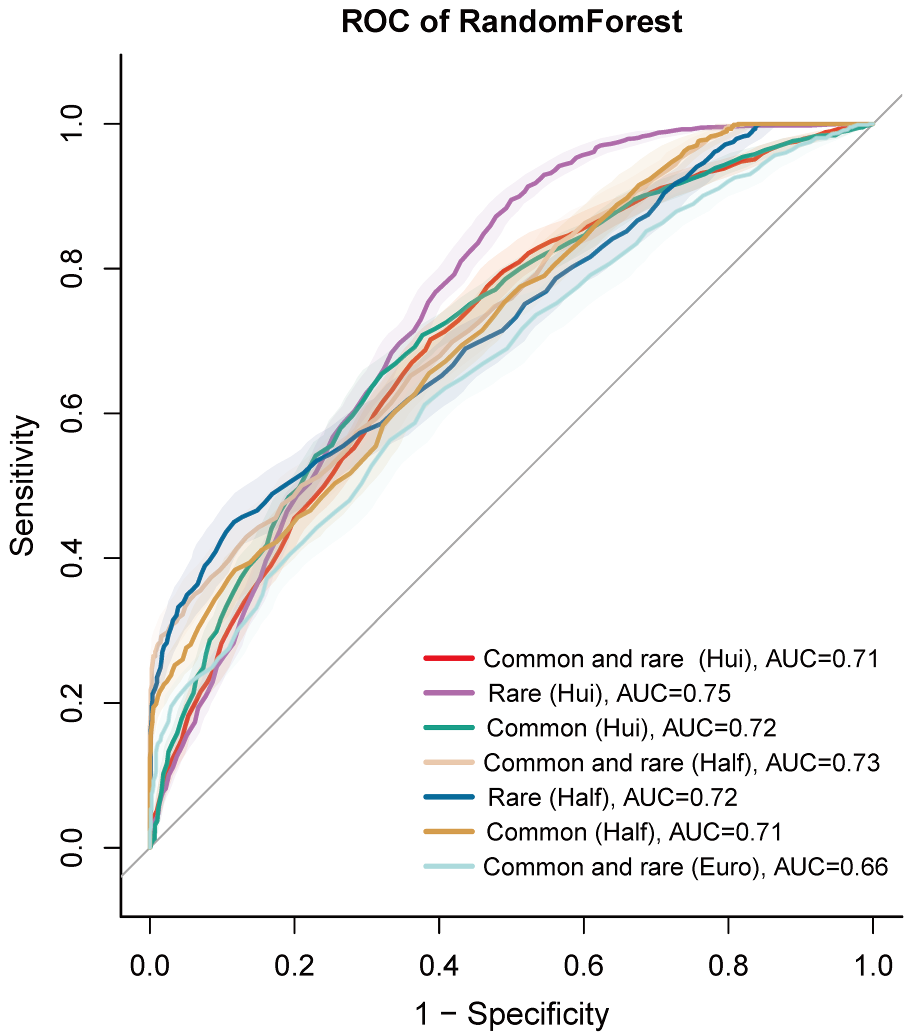

Minor comments:

- The statement on p2 that genes were "validated" in RNA-seq data seems too strong. The associations were not validated, they were just given further biological plausibility.

The sentence was rephrased to read: 'we performed meta- and pathway enrichment analyses to identify novel plausible IBD-causing candidate genes whose biological plausibility were further conferred by bulk RNA sequencing (RNA-seq) and single-cell RNA sequencing (scRNA-seq) analyses.'.

- A TLR4 associations with CD have been described before. Is the TLR4 association in this paper independent of the previously described TLR4 coding variant in CD (rs4986790, described in PMID: 26974007)?

The variant associated with IBD in *TLR4* is rs5031050 in this study, with a P value = 0.048. After conditioning on rs4986790 using GCTA, the new P value was 0.049. Therefore, we conclude that it is independent of rs4986790.

- There seems to be some contradiction in the section on rs574989226/INPP5D. The text states "the most significant variant in INPP5D: rs574989226 (P = 0.011)", but then in the conditional analysis section it states "The significance of rs574989226 only slightly changed after the GCTA-COJO conditional test using our AJ cohort (conditioned, $P = 6.9 \times 10{-}3$; unconditioned, $P = 8.8 \times 10{-}3$ from GCTA-COJO)". Is the unconditional p-value 0.011 or 0.0088?

The difference between the unconditional P values may stem from the different algorithms used in association analyses: the P = 0.011 value comes from the logistic regression model implemented by plink1.9 whereas the unconditional P=0.0088

comes from the GATC running mixed linear model for association analysis. For the sake of consistency, only P values generated from GCTA were used to evaluate the independence of SNPs. We added subscripts to the P value (P_{con} and P_{uncon}) results from conditional tests and removed the previous P values from the text for clarification.

- The authors should provide site and genotype quality scores (including the VQSLOD and VQSR input fields, as well as the missingness, differential missingness, hardy-weinberg p-value, etc) for the variants in Table S11.

We thank the Reviewer for this suggestion. We have updated Table S11 by adding the information mentioned.

- The output given in Supp Tables 13 + 14 are difficult to understand, please fully describe all columns in the table legend. Please also give the input summary statistics for each of the individual datasets.
We thank the Reviewer for this suggestion. We have added illustrative table headers to explain the meaning of each column in the supplementary file. To be consistent with the results of SKAT-O analysis, which were used for the identification of IBD-associated genes, we reported the table of meta-analysis for IBD only (Supplementary Table 15). Also, summary statistics for each dataset have been attached in the supplementary files (Supplementary Tables 13 and 14).

- The legend of Figure S2 incorrectly refers to Figure 2a (presumably this should be Figure 1b).

We have corrected the legend to Figure S2.

Responses to reviewer 2

Using an exome sequencing approach the authors have identified 7 novel IBD-causing genes in 4,974 genetically identified AJ subjects. This is an organized and thoughtful association analysis pipeline followed up with RNASequencing to validate the identified genes from the association analyses. My specific comments include:

We thank the Reviewer for their very helpful and thoughtful comments and suggestions. We have revised the manuscript accordingly.

1) There are many supplementary tables that are not referenced in the results or methods. It is confusing to parse through all these when not cited in the primary body. As an illustration of the confusion - Supplementary Table 2 is the first table one would expect as that is the primary SKAT-O result, this should then connect to the table with the single-SNP results and then followed by the 9 genes identified from the pathways? Please remove tables not referenced or reference them in the submission.

We apologize for the confusion and thank the Reviewer for these helpful suggestions. We have updated all supplementary tables and adjusted their order in the manuscript.

2) Why are single variant tests done in both SKAT-O as well as logistic regression models (reflected in Sup Table 5)? Also I am missing the point of the single variant tests where is is framed as 'To examine the contribution of variants within the significant genes' but then the single variants are evaluated at a GWAS threshold? If this is really to assess the contribution of the single variants to the genes identified through the gene-based approach, then the individual variants should not be penalized for GWAS thresholds.

We thank the Reviewer for this comment. As SKAT performs single variant tests for binary traits using Firth and efficient resampling methods, which only provide *P* values for the variants tested, we employed a logistic regression model to obtain additional information about the effect sizes of the variants, including the odds ratios. We agree that the individual variants should not be penalized for GWAS thresholds for assessing the contributions of single variants. In order to avoid any confusion for readers, we kept the variant-level results from logistic regression tests for variants passing nominal significance ($P < 0.05$).

3) Why only the 9 genes identified with the pathway approach further prioritized. The rationale to the pathway approach was "Since biologically relevant genes may not display genome-wide significance at the gene level due to genetic heterogeneity, we additionally applied pathway enrichment and biological relatedness approaches to identify biologically plausible IBD-causing genes from the SKAT-O significant genes." One would argue that under this rationale the final set of genes for

prioritization should in fact be the union of those identified at exome-wide thresholds (n=15 genes) AND the 9 from pathways, and not limited to only those 9 from the pathway.

We thank the Reviewer for this comment. We performed gene prioritization for all genes with a $P < 0.01$ in SKAT-O analyses, including the exome-wide significant genes and the 9 genes (11 genes in the updated results) identified by all pathway approaches. In fact, we used the results of pathway analyses to perform two different analyses, the first aimed at identifying further plausible IBD genes from the SKAT-O results, whereas the purpose of the other was to prioritize the candidate genes by counting the number of known IBD genes residing in the same pathway as each candidate gene. By following this approach, we were able to provide a list of sorted genes according to their functional proximities to the known IBD genes. The updated results are attached in Supplementary Table 6.

4) Why was GCTA-COJO used for conditional analysis when line level data is available? Would it not be more appropriate to model the SNPs jointly in the specific dataset that rely on summary statistics.

We thank the Reviewer for this important comment. We performed the conditional analyses because we discovered that some of the known IBD genes resided close to the IBD candidate genes found in this study. Therefore, we wanted to see if there were some undiscovered connections between previous IBD loci and the new candidate loci identified in the current study. The results indeed revealed that the leading SNPs in *ICAM1* and *INPP5D* have independent associations to IBD.

5) In the definition of the 'high impact' variant in "These 9 plausible IBD candidate genes harbor 55 high impact variants (Supplementary Table 1 and 11), it would seem that there are genes with only a single variant in the gene-based skat-o analysis. Please add #variants to Supp Table 1. Also why would just the 'top 5 ranking' SNPs be collapsed into a single set? The rationale seems unclear. Would be it more appropriate to consider the cumulative burden across all 9 genes as a single unit without filtering?

We thank the Reviewer for this comment, and agree that obtaining the cumulative burden across all genes is a very reasonable and valuable suggestion. We therefore updated the results following both Reviewers' suggestions to compare the cumulative burden across all genes in the IBD cases and controls. Following a previous method (aggregating variants at different magnitudes of impact), we have tested an extreme situation by aggregating the most significant variants within each IBD candidate gene (if any are present) to check the burden of the variant set. The results have been revised as presented in the following paragraph:

'*These 11 plausible IBD candidate genes harbor a total of 46 high impact variants (Supplementary Table 11). To test the burden of the significant SNPs ($P < 0.05$) located within the IBD candidate genes, we aggregated all significant SNPs from each IBD candidate gene into a single SNP set; the mutation carrier frequency in cases was 15.74% compared to 9.26% in controls, with an odds ratio (OR) of 1.83 ($P = 8.78\times10^{-11}$ by chi-squared test) despite two protective sites that are included in the analyses.*'
Please also note that we checked the cumulative burden across all genes; the results displayed an OR of 1.47 for IBD cases *vs*. controls, with $P = 3.29\times10^{-7}$.

We have added the number of the variants for each gene in the updated Table 1. As can be seen in the table, there are genes with only one variant. Other variants in these genes were too rare to obtain available ORs and p values because they were absent in either cases or controls. Therefore, we did not include the variants without available ORs in the table. Table 1 has been merged into the Supplementary Table 11; furthermore, it has been revised based on the updated results:

Gene	Gene *P* value	Rank	Main SNP in gene	rsID	SNP OR	SNP *P* value	Tested Marker
EGR2	7.19×10^{-3}	11	10:64573941:T:G	rs202183386	2.56 [2.50 - 2.62]	9.05×10^{-3}	3
ICAM1	2.94×10^{-3}	3	19:10395877:C:T	rs142682313	0.43 [0.42 - 0.43]	2.28×10^{-3}	6
INPP5D	2.82×10^{-4}	4	2:233990635:TC:T	rs574989226	2.84 [2.77 - 2.91]	1.07×10^{-2}	5
ITK	3.59×10^{-3}	8	5:156675967:C:T	rs34482255	2.03 [2.00 - 2.07]	1.16×10^{-2}	4
LRRK2	2.53×10^{-4}	6	12:40734202:G:A	rs34637584	2.50 [2.46 - 2.54]	3.51×10^{-4}	28
NOD2	3.03×10^{-9}	2	16:50745656:G:A	rs104895438	3.34 [3.28 - 3.40]	1.68×10^{-5}	26

PDGFD	5.04×10^{-3}	17	11:103797657:C:T	rs151199614	2.31 [2.26 - 2.35]	4.46×10^{-3}	5
TLR4	4.79×10^{-3}	1	9:120475431:T:A	rs5031050	0.27 [0.26 - 0.28]	4.88×10^{-2}	4
VDR	8.07×10^{-3}	5	12:48272845:G:A	rs147496897	2.97 [2.90 - 3.05]	6.30×10^{-3}	3

6) Please clarify the rationale to picking 268 differentially expressed genes because that number aligns with 268 skat-o identified genes with p<0.01. This seems arbitrary. RNASeq generally has the ability to identify more differential signal that association tests, and as such should not be held to a 'count' of top genes to align with the number passing the skat-o significance levels.
I could not understand this part either.

We thank the Reviewer for this important comment. We agree that using a set of genes with p < 0.01 from the SKAT-O results for prioritizing candidate genes from RNA-seq might appear arbitrary. The top genes derived from genetic association tests and differential expression are indeed not comparable as they were obtained from genome-level and transcriptome-level analyses, respectively. Therefore, only using the same number of top genes to repeat the pathway analysis could be obscure. In response to the Reviewer's comment, we performed another IPA analysis by adding differential expression values to the top genes from the SKAT-O test. We found that the IPA results weighted by gene expression information were the same as with our original IPA analysis.

7) Please address significance thresholds. Early in results the exome-wide Bonferroni threshold is used to define 15 genes, this then switched to those with p<0.01 for the pathway approach to identify 9. However in the conclusion genes with p<0.01 are defined as 'significant'.

We are grateful to the Reviewer for pointing this out. We set a relaxed threshold for statistical significance in the pathway and enrichment analyses by selecting genes with a P < 0.01 in SKAT-O. Our intention in following such an approach was to reduce the effect of genetic heterogeneity and to capture other possible disease-relevant genes. In the updated manuscript, we make mention of the reason for using P < 0.01 for the pathway analysis and rephrased the sentences in the conclusion: '*Since biologically-relevant genes might not display genome-wide significance at the gene level due to genetic heterogeneity, we additionally applied pathway enrichment and biological relatedness approaches to identify biologically plausible IBD-associated genes that we obtained from the SKAT-O results. The significance cut-off in the AJ IBD SKAT-O test was relaxed to P < 0.01 to capture other possible IBD-associated candidates.*'

REVIEWER COMMENTS

Reviewer #1 (Remarks to the Author):

The authors have carried out a substantial revision of the paper, and have addressed the majority of the issues I have raised. The additional filtering, QC and association controls seem to have cleaned up the data substantially, and the control-vs-control and missense analyses seem robust.

I have a few small comments on the new version of the manuscript:

- While I agree that replacing a single-variant PheWAS with a SKAT based PheWAS will reduce the chance of confounding due to common variants, I still believe that the potential for bleed-through signal still exists (a common variant signal can still bleed through into a burden result, and this does not seem to have been controlled for). I think this is most likely to be the case in the ICAM1 T1D association, as it is known that ICAM1 variants are in LD with TYK2 variants (which has previously made disentangling the two difficult), and T1D is a disease that is known to be associated with TYK2 variants. In a recent UK Biobank PheWAS burden analysis (PMID:31866045), the authors used SKAT-O to check their rare burden associations associations were independent of known common variation (and found that they were). I would encourage the authors to use this same approach in their PheWAS in cases where a GWAS hit for that phenotype is nearby.

- In Figure 3E, the confidence intervals on the ROC curve are useful, but it would also be useful if the authors also provided a 95% confidence interval on the estimated AUCs in the legend.

- For the authors information, according to the text of de Lange et al, the genome-wide summary statistics files are available from here:
ftp://ftp.sanger.ac.uk/pub/project/humgen/summary_statistics/human/2016-11-07/

I do not necessarily recommend re-running the PRS using these files (the Liu et al data is be fine), but they are available the authors wish to use them.

Reviewer #2 (Remarks to the Author):

The resubmission is greatly improved, and the QC issues pointed out by Reviewer #1 appear to have identified and addressed some major artifacts and potential false positives. There are two pending queries from my initial review where I do not find the response sufficient.

Prior query:Why was GCTA-COJO used for conditional analysis when line level data is available? Would it not be more appropriate to model the SNPs jointly in the specific dataset that rely on summary statistics. The authors simply clarify why conditional analysis was used. That is not the question being raised. My understanding of COJO is that is uses summary statistics to perform the conditional analysis. Why was this the choice of analysis approach when the line-level data on both SNPs being examined are directly available?

Prior query: Please clarify the rationale to picking 268 differentially expressed genes because that number aligns with 268 skat-o identified genes with $p<0.01$. This seems arbitrary. RNASeq generally has the ability to identify more differential signal that association tests, and as such should not be held to a 'count' of top genes to align with the number passing the skat-o significance levels. The authors respond with agreement that this is an obscure choice. However I see once again that 127 genes were identified with a $p<0.01$ in the skat-o. And, again 127 'highly differential' genes from RNASeq were moved forward to IPA. First - I assume it was the top 127 DEGs, but once again, I am confused as to why the number of genes was held to the number crossing the $p<0.01$ from skat-o. For IPA on the

RNASeq DEGs, why would an appropriate FDR set of genes not be moved forward to IPA?

Reviewer #1

The authors have carried out a substantial revision of the paper, and have addressed the majority of the issues I have raised. The additional filtering, QC and association controls seem to have cleaned up the data substantially, and the control-vs-control and missense analyses seem robust.

I have a few small comments on the new version of the manuscript:
- While I agree that replacing a single-variant PheWAS with a SKAT based PheWAS will reduce the chance of confounding due to common variants, I still believe that the potential for bleed-through signal still exists (a common variant signal can still bleed through into a burden result, and this does not seem to have been controlled for). I think this is most likely to be the case in the ICAM1 T1D association, as it is known that ICAM1 variants are in LD with TYK2 variants (which has previously made disentangling the two difficult), and T1D is a disease that is known to be associated with TYK2 variants. In a recent UK Biobank PheWAS burden analysis (PMID:31866045), the authors used SKAT-O to check their rare burden associations associations were independent of known common variation (and found that they were). I would encourage the authors to use this same approach in their PheWAS in cases where a GWAS hit for that phenotype is nearby.

We thank Reviewer #1 for this important comment. Following the Reviewer's suggestion, we reached out to the authors of the published work (PMID:31866045) in order to obtain the details of the conditional analysis for the SKAT-O test as they were not mentioned in their original paper. We then used the same method to validate the independence of the rare burden associations. We also used this opportunity to repeat the gene-level PheWAS using the most recent medical records of the BioMe BioBank participants, which identified similar significant associations to those in the previously reported PheWAS.
Specifically, we first extracted all common variants within ±100 kbp of significant rare variants ($p < 0.05$) from the imputed array data of BioMe, and then performed single variant tests with SKAT-O using the same parameters as the gene level test (1:10 case control ratio, PC1 and PC2 as covariates), and selecting the most significant common variant from the results. Lastly, we repeated conditional gene-level tests for the significant PheWAS associations using the common variant allele count (0-1-2) as an additional covariate following the suggestion of the authors of PMID 31866045. The conditional analyses were applied to the following associations: K50.00 - *NOD2*, K50,90 - *NOD2*, K51.00 - *ICAM1*, E10.9 - *ICAM1*, G20 - *LRRK2*. The results showed that all associations remained significant after the conditional analysis. Regarding *ICAM1*, we found that the most significant common variant for the E109 – *ICAM1* association was in the *MRPL4* gene, whereas *TYK2* harbored the second most significant common variant around *ICAM1*. We therefore performed conditional analyses for E10.9 – *ICAM1* on both common variants residing in *MRPL4* and *TYK2* respectively. We found that all associations remained significant after the conditional analysis (Supplementary Table 18), indicating that the rare variant-derived associations in the PheWAS analyses are independent of neighboring common variants.
The following related content was added to the Supplementary Results:

'*We futher performed conditional analysis to evaluate whether the signals of collapsed rare variants in PheWAS were independent of the nearby common variant association signals (±100 Kbp up and down stream) following previous study*[4]*. To identify most significant nearby variants, we first extracted all common variants in 100kbp nearby of significant rare variants ($p < 0.05$) from imputed array data of the Mount Sinai BioMe BioBank, then performed single variant tests with SKAT-O using the same parameters as the gene level test (Method). All associations remained significant after the conditional analysis (Supplementary Table 18). Because TYK2 is a known type 1 diabetes-associated gene, which is located in proximity of ICAM1, we also performed a conditional analysis for the most significant common variant of TYK2 located in ±100 Kbp up and down stream of ICAM1. The results showed that the ICAM1-Type 1 diabetes mellitus association was still signficant after conditional test (Supplementary Table 18).*'
4. Zhao, Z., et al. UK Biobank whole-exome sequence binary phenome analysis with robust region-based rare-variant test. The American Journal of Human Genetics 106.1 (2020).

Supplementary Table 18.

Conditional analysis for gene-level PheWAS results on the nearby significant common variants around the rare variants												
Code Interpretation	ICD10 code	Cases	Controls	Gene	most_sig_rare_var	most_sig_rare_P	most_sig_common_var	Gene_most_sig_common_var	most_sig_common_P	P.value (SKAT-O)	conditional_P (SKAT-O)	Note
Type 1 diabetes	E109	444	4440	*ICAM1*	rs14268231	0.000512	rs113197610	*MRPL4*	0.000899	0.00091	0.02142573	
Type 1 diabetes	E109	444	4440	*ICAM1*	rs14268231	0.000512	rs280516	*TYK2*	0.002585	0.00091	0.000997201	*
Ulcerative (chro	K5100	120	1200	*ICAM1*	rs14613432	0.041964	rs12720356	*TYK2*	0.020118	0.027109	0.02647939	
Crohn's disease	K5000	180	1800	*NOD2*	rs61747625	0.000178	rs2066847	*NOD2*	3.46E-08	2.63E-05	1.37E-05	
Crohn's disease,	K5090	328	3280	*NOD2*	rs61755272	0.003115	rs2066847	*NOD2*	4.46E-09	0.00179	5.22E-04	
Parkinson's dise	G20	335	3350	*LRRK2*	rs34637584	2.41E-09	rs564036500	*LRRK2*	0.00017	7.36E-12	5.17E-07	
*: The 2nd most significant common variant for E109 - ICAM1												

The table was captured as a figure from the Supplementary Results to fit the width of this document.

- In Figure 3E, the confidence intervals on the ROC curve are useful, but it would also be useful if the authors also provided a 95% confidence interval on the estimated AUCs in the legend.

We thank the Reviewer for this helpful suggestion. We have added the 95% confidence interval of the estimated AUCs to the legend. The descriptions in the main text have been modified accordingly. Please see updated figure:

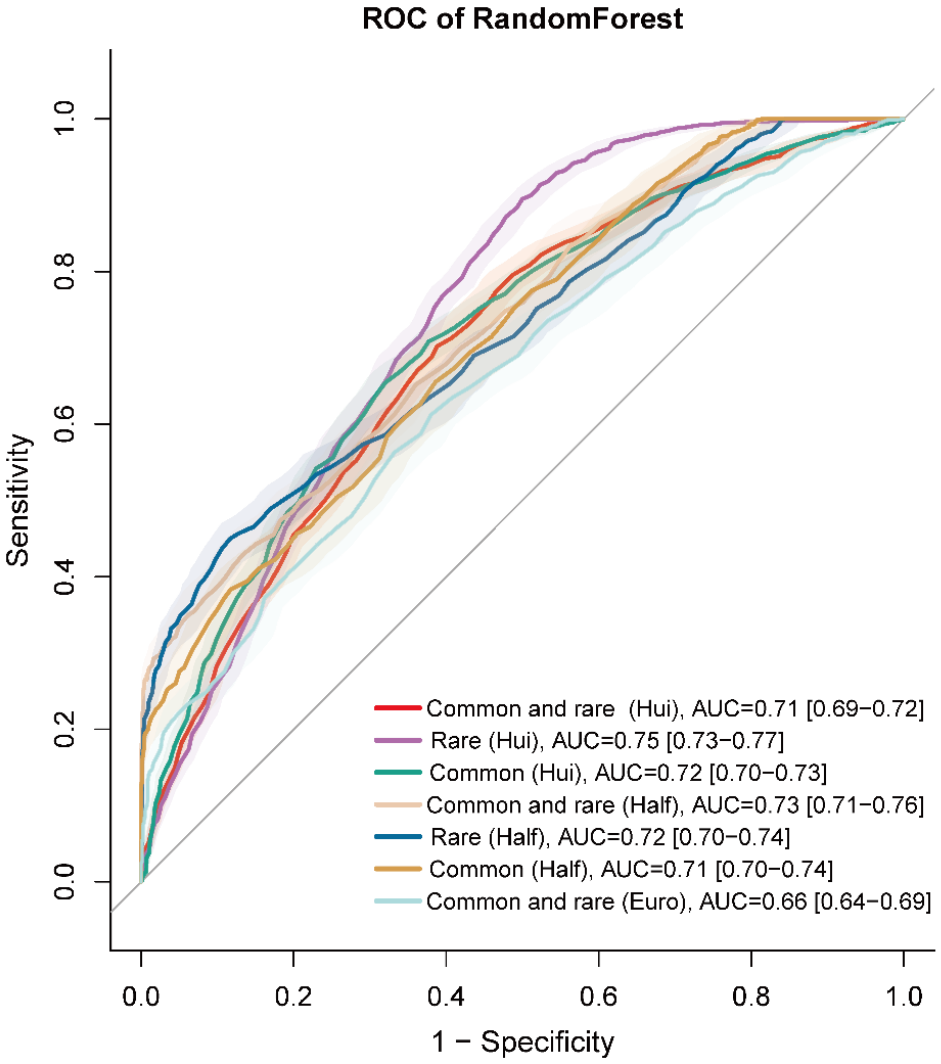

- For the authors information, according to the text of de Lange et al, the genome-wide summary statistics files are available from here:
ftp://ftp.sanger.ac.uk/pub/project/humgen/summary_statistics/human/2016-11-07/
I do not necessarily recommend re-running the PRS using these files (the Liu et al data is be fine), but they are available the authors wish to use them.

We thank the Reviewer for providing this useful data resource. We will take advantage of it in our future IBD projects.

Reviewer #2

The resubmission is greatly improved, and the QC issues pointed out by Reviewer #1 appear to have identified and addressed some major artifacts and potential false positives. There are two pending queries from my initial review where I do not find the response sufficient.

Prior query:Why was GCTA-COJO used for conditional analysis when line level data is available? Would it not be more appropriate to model the SNPs jointly in the specific dataset that rely on summary statistics. The authors simply clarify why conditional analysis was used. That is not the question being raised. My understanding of COJO is that is uses summary statistics to perform the conditional analysis. Why was this the choice of analysis approach when the line-level data on both SNPs being examined are directly available?

We apologize for not fully addressing these two queries from Reviewer #2. In this revision, we have attempted to better address them. If we have understood the notion of ‘line-level data’ correctly, we assume that the Reviewer was suggesting that we run a joint association analysis to check whether the variants are associated with IBD based on summary statistics, instead of running a conditional test only. We were previously only concerned about the independence of the *ICAM1* lead variant, and therefore we only performed a conditional analysis on the neighboring IBD-associated variants of the *ICAM1* variant. In this revision, we have appended the joint association analysis results to Supplementary Table 9. The *ICAM1* variant rs142682313 remained as an IBD-associated variant. Among the 3 variants in *TYK2*, which were reported as IBD-associated sites, only rs12720356 was an IBD associated-variant in our AJ cohort.
The related context has been corrected to:
‘*Since ICAM1 is located within 100kb of TYK2 (a gene known to be associated with IBD pathogenesis*[15]*, we sought to determine whether the ICAM1 lead variant (rs142682313, OR=0.4, P = 7.16×10*$^{-04}$*) was conditionally independent of IBD-associated sites in TYK2. To this end, we performed Genome-wide Complex Trait joint and conditional analyses (GCTA-COJO)*[16] *with the ICAM1 lead SNP and three IBD-associated sites in TYK2, both of which suggested that the ICAM1 IBD variants act independently of the TYK2 variants. One of the TYK2 IBD variants, rs12720356, remained as an IBD-associated variant in the AJ cohort based on joint association analysis* (Supplementary Table 8 and 9).’

Supplementary Table 9 (joint association analysis part)

Joint association analysis for *ICAM1* lead variants and 3 *TYK2* variants

Chr	SNP	RS ID	Gene	Bp	Effect Allele	Frequency of the effect allele	Effect size (Original)	Standard error (Original)	P value (Original)	Effect size (Joint analysis)	Standard error (Joint analysis)	P value (Joint analysis)
19	19:10395877:C:T	rs142682313	*ICAM1*	10395877	T	0.0090026	-0.18398	0.051738	0.000377	-0.1774	0.051838	0.000621
19	19:10463118:G:C	rs34536443	*TYK2*	10463118	C	0.0319583	-0.02787	0.027973	0.319054	-0.02054	0.028055	0.463986
19	19:10464843:G:A	rs35018800	*TYK2*	10464843	A	0.00182	0.041541	0.114214	0.716074	0.043238	0.114219	0.705021
19	19:10469975:A:C	rs12720356	*TYK2*	10469975	C	0.104441	0.054636	0.015974	0.000626	0.051771	0.016051	0.001258

The table was captured as a picture from the supplementary file to fit the width of the document for better view.

Prior query: Please clarify the rationale to picking 268 differentially expressed genes because that number aligns with 268 skat-o identified genes with p<0.01. This seems arbitrary. RNASeq generally has the ability to identify more differential signal that association tests, and as such should not be held to a 'count' of top genes to align with the number passing the skat-o significance levels. The authors respond with agreement that this is an obscure choice. However I see once again that 127 genes were identified with a p<0.01 in the skat-o. And, again 127 'highly differential' genes

from RNASeq were moved forward to IPA. First - I assume it was the top 127 DEGs, but once again, I am confused as to why the number of genes was held to the number crossing the p<0.01 from skat-o. For IPA on the RNASeq DEGs, why would an appropriate FDR set of genes not be moved forward to IPA?

We are grateful to Referee #2 for pointing this out. We apologize for the misunderstanding and for not fully addressing the Reviewer's query. Indeed, we agree with the Reviewer that this was an obscure choice. We also join the Reviewer in deprecating the pathway analysis performed by picking the differentially expressed genes aligning with the number of genes with p<0.01 in the SKAT-O test. Instead, we performed IPA analyses for the top genes identified from SKAT-O by assigning weight (fold change and P-value obtained from the RNA-seq analyses) to each gene, which was described in the response. However, we inadvertently failed to correct the related part of the main text apart from the number. We have now updated this part of the main text to read as follows:
'*We performed pathway analysis for the 127 significant genes (P < 0.01) identified by SKAT-O and weighing the significant genes based on their log fold change and p values obtained from bulk RNA-seq analyses. Among the results of related 'Disease and Disorder' analysis by IPA, the 'Cancer', 'Organismal Injury and Abnormalities' and 'Gastrointestinal Disease' were the top 3 mostly related disorders.*'
As the Reviewer mentioned, the conventional means of performing downstream analysis for RNA-seq was to select a set of DEGs by cutoffs of FDR and logFC, before proceeding to pathway analyses. In this study, we primarily conducted genetic analyses, whereas the bulk RNA-seq data were used to determine whether the candidate genes are also differentially expressed between IBD cases and unaffected controls. Thus, we did not perform additional analyses at the transcriptome level. Our aim in the first version of the manuscript was to check whether there was some consensus between genomic and transcriptomic results by analyzing the same number of gene sets from these studies respectively. Here, we have followed the Reviewer's suggestion and removed them from our study. We thank the Reviewer again for pointing out this issue.

REVIEWERS' COMMENTS

Reviewer #1 (Remarks to the Author):

The authors have address all of my outstanding issues. I thank them for all their hard work in responding to my comments.
